# Visual trends and hot research on the relationship between intestinal microbiota and major lipids: a bibliometric analysis

**DOI:** 10.3389/fmicb.2024.1361439

**Published:** 2024-04-30

**Authors:** Weiming Sun, Keqi Wan, Jiawei Gui, Guoqiang Jin, Lang Shuai

**Affiliations:** ^1^Department of Rehabilitation Medicine, The First Affiliated Hospital, Jiangxi Medical College, Nanchang University, Nanchang, China; ^2^The First Clinical Medical College, Jiangxi Medical College, Nanchang University, Nanchang, China; ^3^Postdoctoral Innovation Practice Base, The First Affiliated Hospital, Jiangxi Medical College, Nanchang University, Nanchang, China; ^4^HuanKui Academy, Jiangxi Medical College, Nanchang University, Nanchang, China; ^5^Department of Health Management, The First Affiliated Hospital, Jiangxi Medical College, Nanchang University, Nanchang, China

**Keywords:** intestinal microbiota/gut microbiota, lipids, lipid metabolism, bibliometric analysis, trends, CiteSpace, hotspot

## Abstract

**Objective:**

The association between intestinal microbiota and lipids has garnered significant scholarly interest. This study analyzes pertinent literature on intestinal microbiota and lipids to offer scientific guidance for future advancements and research directions.

**Methods:**

Articles focusing on intestinal microbiota and lipids were obtained from the Web of Science Core Collection (WoSCC). Following a rigorous screening process, 12,693 articles were included in the study. The collected data was processed comprehensively and visually analyzed using various academic tools such as CiteSpace, VOSviewer, R software, and Scimago Graphica.

**Results:**

The field of intestinal microbiota and its relationship with major lipids has witnessed a significant surge in scholarly attention, as indicated by the upward trend observed in related articles. Among countries, China had emerged as the leading contributor in publication output, with Chinese Acad Sci being the most prolific institution in this field. Notably, Nutrients and Nature were the prominent journals that published many articles and garnered the highest number of co-citations. Scholars have widely recognized Patrice D Cani's notable contributions in this field. Current research endeavors have focused on obesity, insulin resistance, metabolism, growth performance, the gut-brain axis, and others.

**Conclusions:**

Our analysis identified four primary research trends: “biochemical pathways,” “exploration of diseases,” “intervention and effect,” and “health and diet.” Future scholars must devote more attention to intestinal microbiota and major lipids to advance our understanding of human health.

## 1 Introduction

The interplay between the intestinal microbiota and major lipids has garnered considerable attention in recent years, owing to its implications for human health and disease. The human gastrointestinal tract harbors trillions of microorganisms, collectively known as the gut microbiota or intestinal flora (Liu et al., 2021). These microorganisms are primarily involved in various gut-related functions including regulation of intestinal motility, food digestion, nutrient absorption, and maintenance of intestinal integrity (Młynarska et al., 2022). Furthermore, the gut microbiota forms a complex ecosystem that intricately regulates multiple metabolic processes in the host, encompassing lipid metabolism, energy homeostasis, and glucose metabolism (Schoeler and Caesar, 2019).

Lipids, mainly including triglycerides, phospholipids, and cholesterol, are ubiquitous in the human body and play an important role as a medium for energy storage and cell signaling pathways (Raman et al., 2017). Lipid metabolism disorders can lead to various associated diseases, including but not limited to obesity, atherosclerosis, cardiovascular disease, diabetes, and non-alcoholic liver disease (Madsen and Ramosaj, 2021). The regulation of lipid metabolism primarily relies on nutrients such as sugars and fatty acids. Numerous studies have demonstrated the correlation between lipid levels and gut microbiota, highlighting the pivotal role of research on intestinal flora in promoting human health and preventing and intervening in related diseases (Schoeler and Caesar, 2019).

Currently, extensive scholarly research had been conducted to explore the relationship between lipids and gut microbiota. However, the majority of these studies had focused on specific aspects, lacking a comprehensive analysis. To enhance the understanding of the intricate connection between intestinal flora and prominent lipids, we conducted a pioneering bibliometric analysis encompassing various articles in this domain. The scope of this study encompasses the analysis of fundamental lipid components in the human body, including triglycerides, phospholipids, cholesterol, and other major lipid species (Cham, 1978). Bibliometric analysis is a widely utilized scientific and quantitative research method for analyzing publications. This approach identifies knowledge networks, research hotspots, and emerging trends in a specific field (Zhang and Tao, 2023). By employing bibliometric tools, we conducted a systematic and scientific analysis of publications on intestinal flora and lipids, focusing on countries, institutions, authors, journals, keywords, and co-citation literature. This analysis aims to comprehensively understand the relationship between intestinal flora and lipids, uncover research hotspots, and shed light on future trends in this field. Consequently, this study provides a solid foundation and invaluable references for scholars in this domain.

## 2 Methods

### 2.1 Data collection strategy

**Figure 1:**
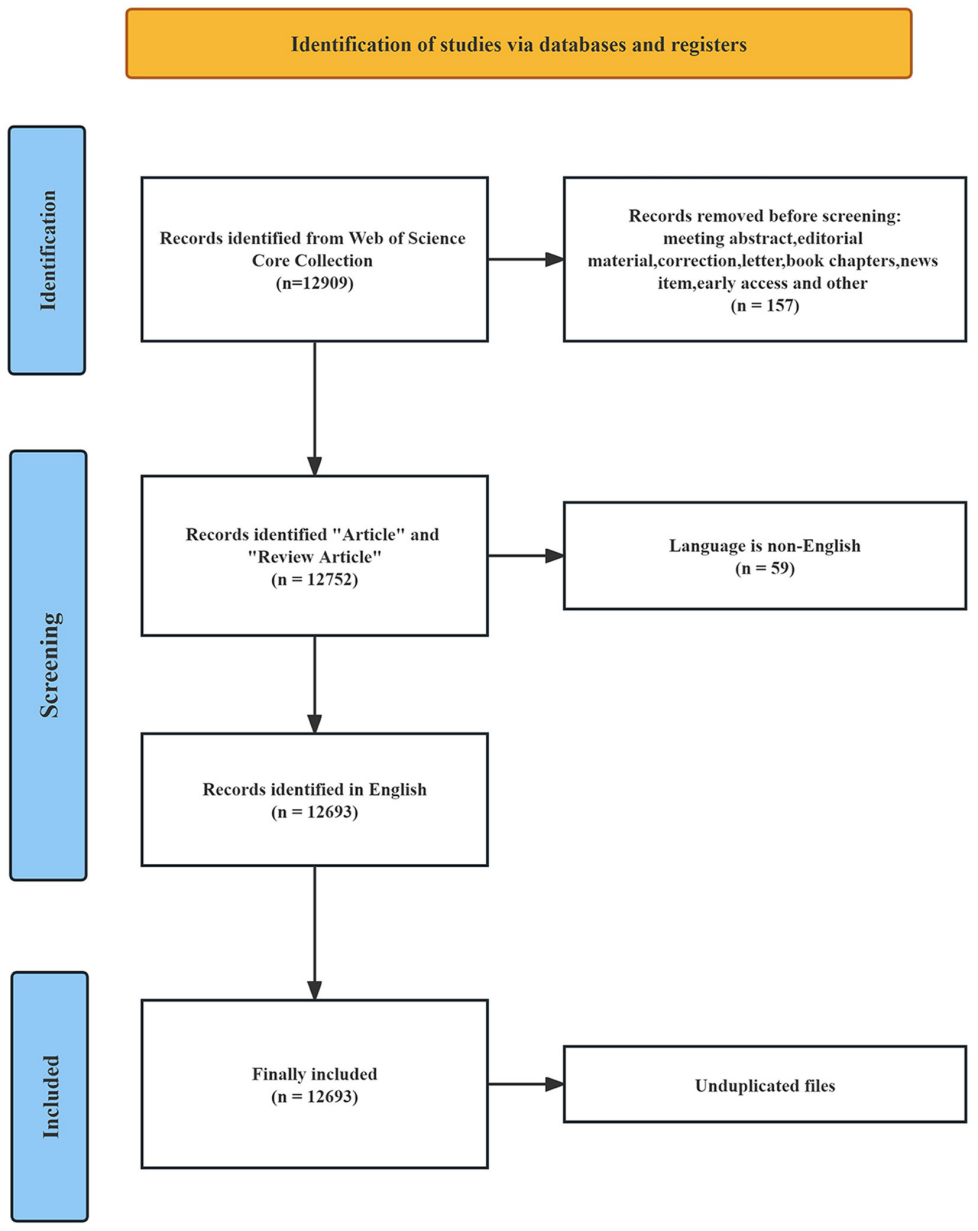
The flowchart of methods included in the study.

The literature data was obtained from the Web of Science Core Collection (WoSCC) at Nanchang University and was fully collected on November 18, 2023. The WoSCC database is a comprehensive information service that facilitates literature search across various disciplines, including the humanities, natural sciences, social sciences, and arts sciences. It encompasses a vast range of scholarly resources, such as journals, patents, books, conference proceedings, and online materials. Being universally recognized, this database serves as a pivotal tool for researchers worldwide, providing extensively indexed publications that form an integral component of the research process (Sun et al., 2022). Considering its reliability and wide-ranging coverage, we selected the WoSCC database as our data source for this study. The search strategy was as follows: TS = (“intestinal flora” OR “gut flora” OR “bowel flora” OR “intestinal microflora” OR “gut microflora” OR “bowel microflora” OR “intestinal microbiota” OR “gut microbiota” OR “bowel microbiota” OR “intestinal tract bacteria” OR “gut bacteria” OR “bowel bacteria”) AND TS = (“lipid” OR “lipids” OR “fatty acid” OR “plasma fatty acid” OR “cholesterol” OR “plasma cholesterol” OR “triglyceride” OR “plasma triglyceride” OR “phospholipid” OR “plasma phospholipid” OR “lipoprotein” OR “glycolipids” OR “sterol” OR “hyperlipidemia” OR “hypertriglyceridemia” OR “hypercholesterolemia”). The search time range was from January 1, 1981 (WoSCC creation time) to November 18, 2023. As depicted in Figure 1, a total of 12,909 articles were obtained through a scientific search. After excluding 157 non-article types, 12,693 English articles remained. Through manual screening and software review, 12,693 documents that met the criteria were eventually included.

### 2.2 Data analysis

According to our requirements and the specific features of each analysis software, we selected CiteSpace (version 6.1.R6), VOSviewer (version 6.1.19), R software (version 4.2.1), and Scimago Graphica (version 1.0.35) as the data analysis tools.

CiteSpace is an analysis software via Java application created by Dr. Chaomei Chen (Synnestvedt et al., 2005). Its uniqueness and influence enable bibliometrics' automation, visualization, and intelligence, thus offering guidance for various types of analysis. We used CiteSpace to make a collaborative network map of authors and cited authors, a dual map of journals, and an analysis of keywords and co-cited references. The time slice was set to 1, while other parameters remained unchanged per the system settings. The node type could be selected based on specific requirements.

VOSviewer is a computer program that can produce bibliometric maps (van Eck and Waltman, 2010). It has three map forms: network visualization, overlay visualization, and density visualization. Using data from VOSviewer, we used Scimago Graphica to create a correlation graph of the top 30 countries by volume.

Bibliometrix in R software is a robust software package specifically designed for conducting quantitative research in bibliometrics and scientometrics (Jiang et al., 2023). We utilized it for generating maps representing various countries, analyzing trends in journal publications, and conducting scientific keyword analysis to enhance the intuitiveness of the findings with a more scholarly touch.

## 3 Results

### 3.1 General information on publications

**Figure 2:**
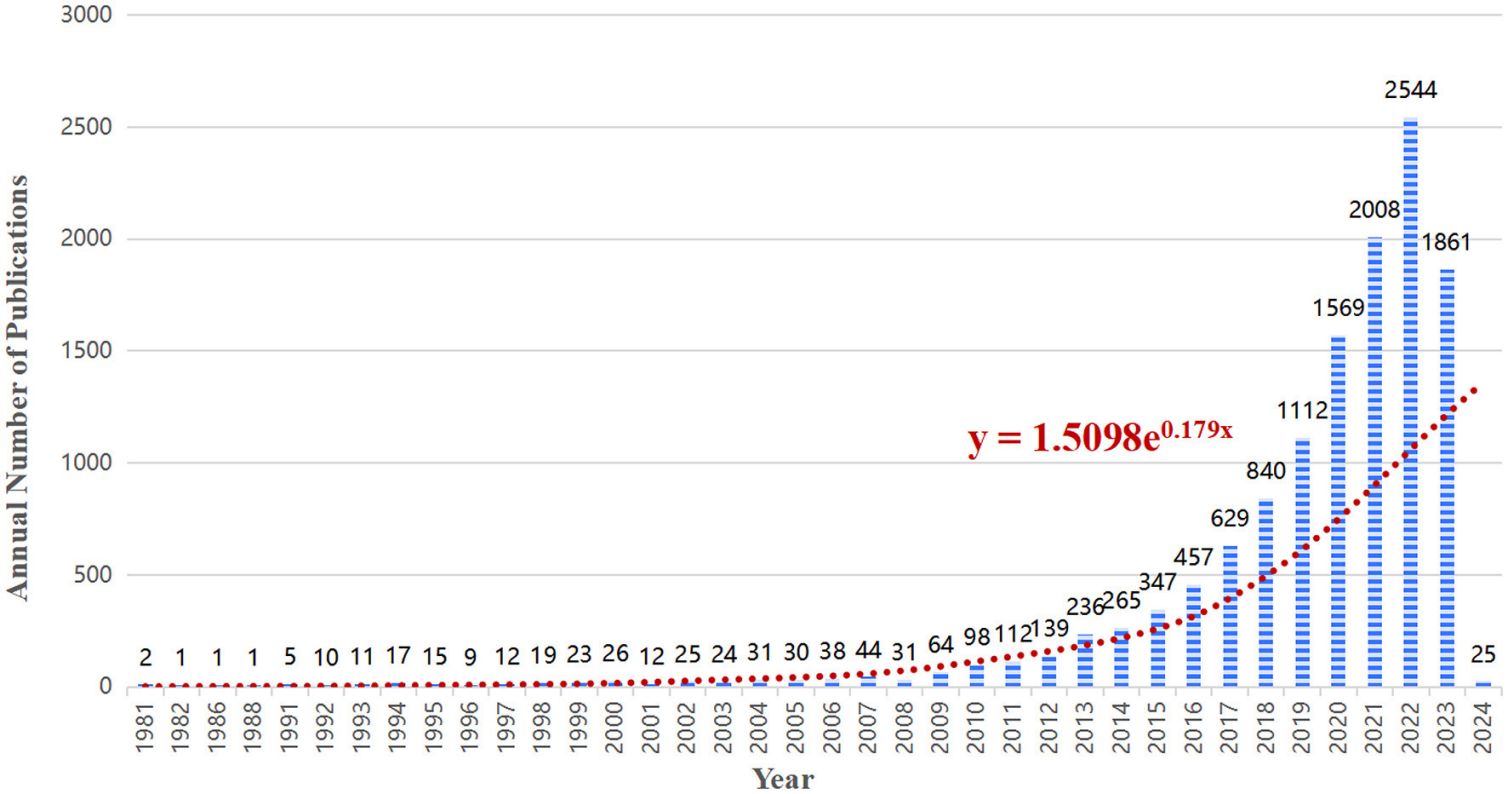
Trends in the number of publications in the field of intestinal microbiota and lipids.

A total of 12,693 articles were included in our bibliometric analysis. Overall, the number of articles on the lipids and gut microbiota field is rising and developing rapidly, indicating that more people are paying more attention to this field. As shown in Figure 2, from 1981 to 2010, the annual publication volume was relatively flat, and no more than 100 papers were published. Since 2011, the annual number of published articles has exceeded 100, and the growth rate is fast, reaching a peak of 2,544 published articles in 2022.

**Figure 3:**
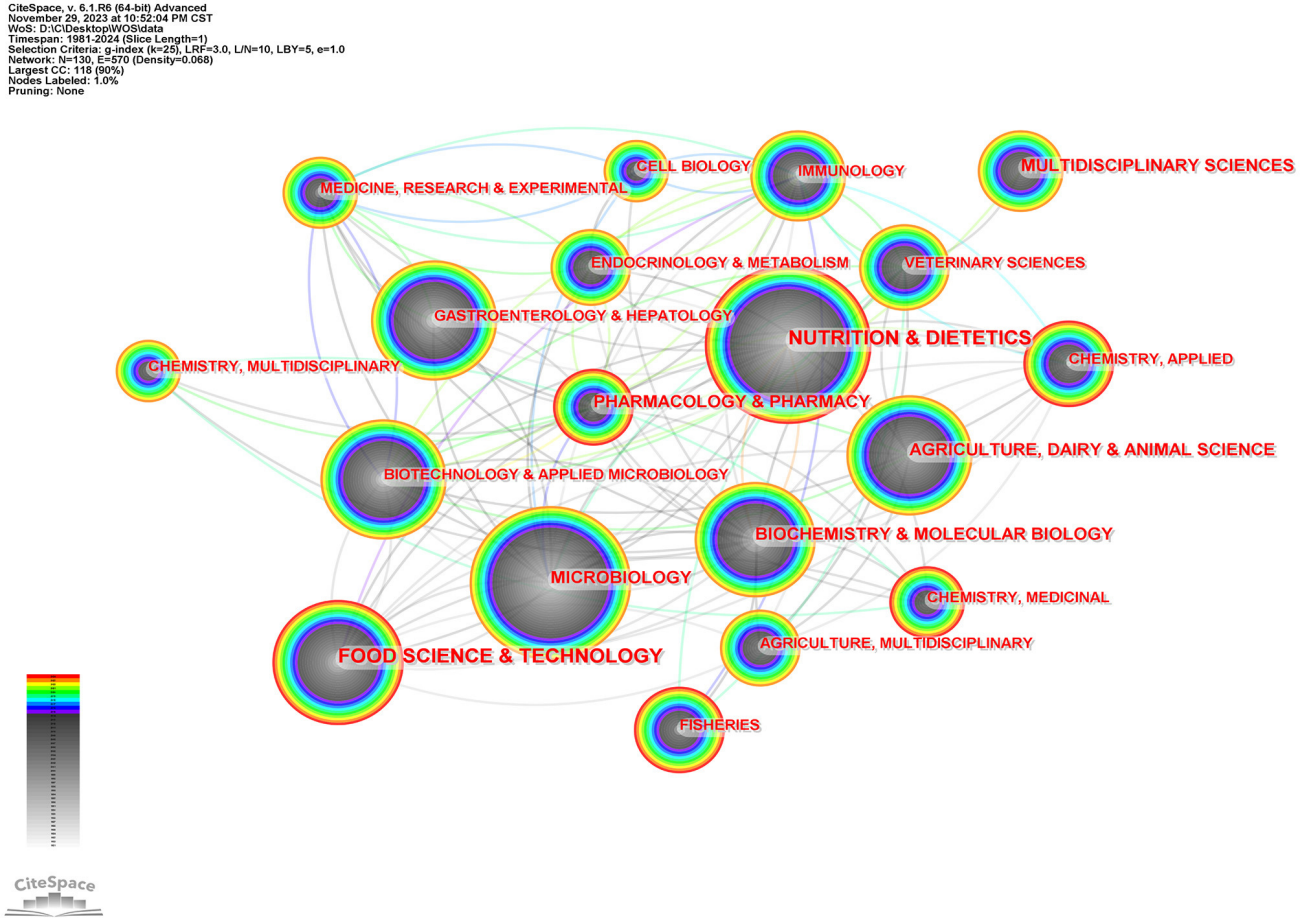
Cooperation networks of categories via CiteSpace.

Figure 3 is the subject category network diagram made by CiteSpace, in which one node represents a subject category. The larger the node, the more articles related to this subject. The lighter and darker the color, the older the year. One hundred and thirty nodes were found in the process of software calculation, indicating that this field has the development of disciplinary diversity. For intestinal flora and lipid research subjects in nutrition and Nutrition & Dietetics, Microbiology, Food Science & Technology, Agriculture, Dairy & Animal Science, Gastroenterology& Hepatology, et al.

### 3.2 Analysis of countries and institutions

**Figure 4:**
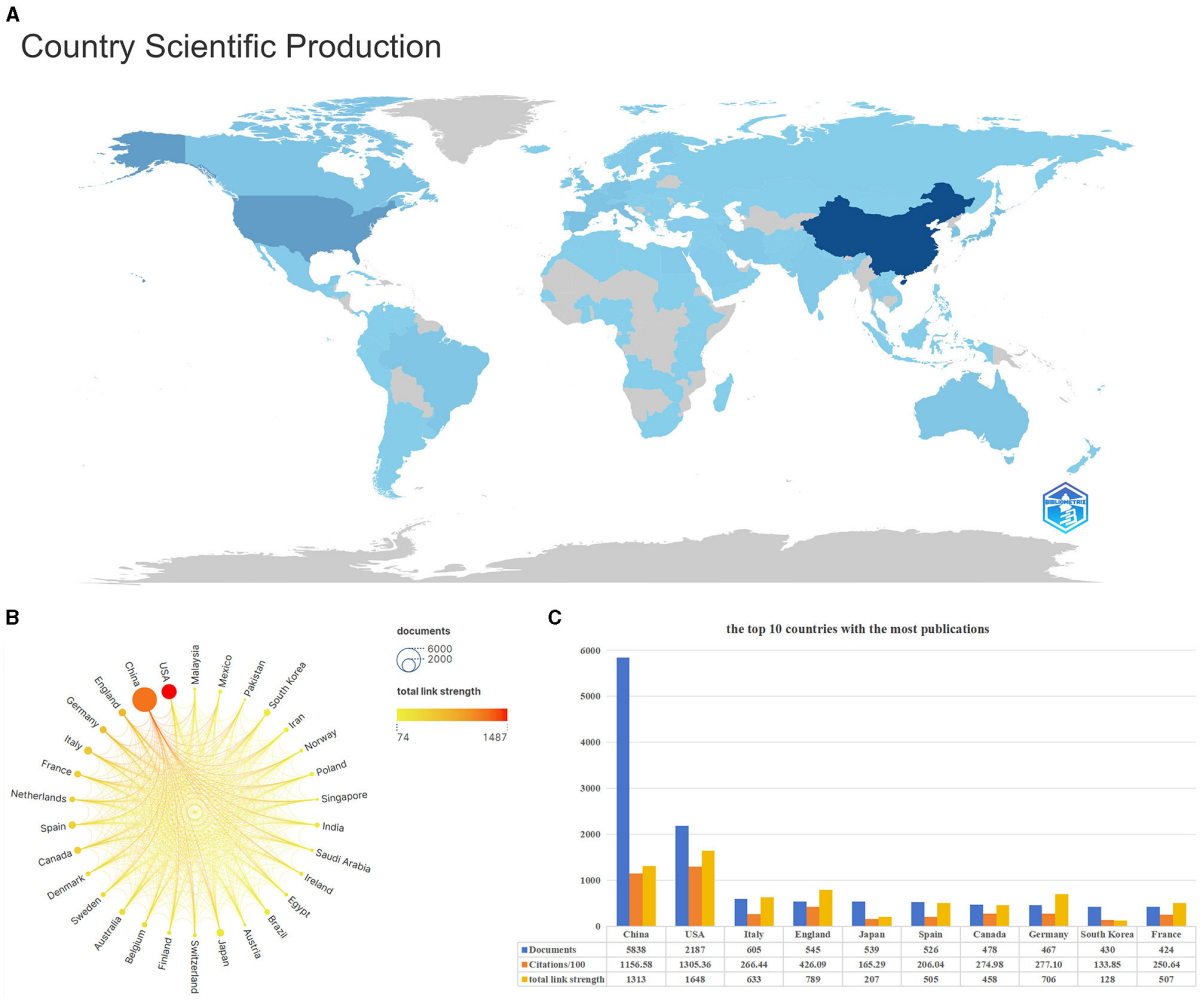
Description of relevant national publications. **(A)** the visual map of national publications, the darker the color, the greater the number of publications in the location. **(B)** Scientific cooperation among the nations of the world. **(C)** The information bar chart of the top 10 countries with the highest number of publications.

Figure 4A is a visual map of a country's publications. The darker the color, the greater the number of publications in that country. China, the USA, and Italy are the top three countries with the most publications. Figure 4B is a national scientific collaboration map using Scimago Graphica and VOSviewer. It compares the number of publications and the link strength between the top 30 countries. China and the USA have published far more articles in this field than others. Although China published more articles than the USA, it had fewer connections to other countries. Figure 4C shows the number of posts, citations, and total connection strength for the top ten most prolific countries. In terms of the number of articles published, China (5,838) was far ahead of other countries, followed by the USA (2,187), Italy (605), England (545), Japan (539), and other countries. The number of articles cited and the total connection strength were higher in the USA than in other countries, showing the importance of American authors in this field and the close cooperation with scholars from other countries.

**Figure 5:**
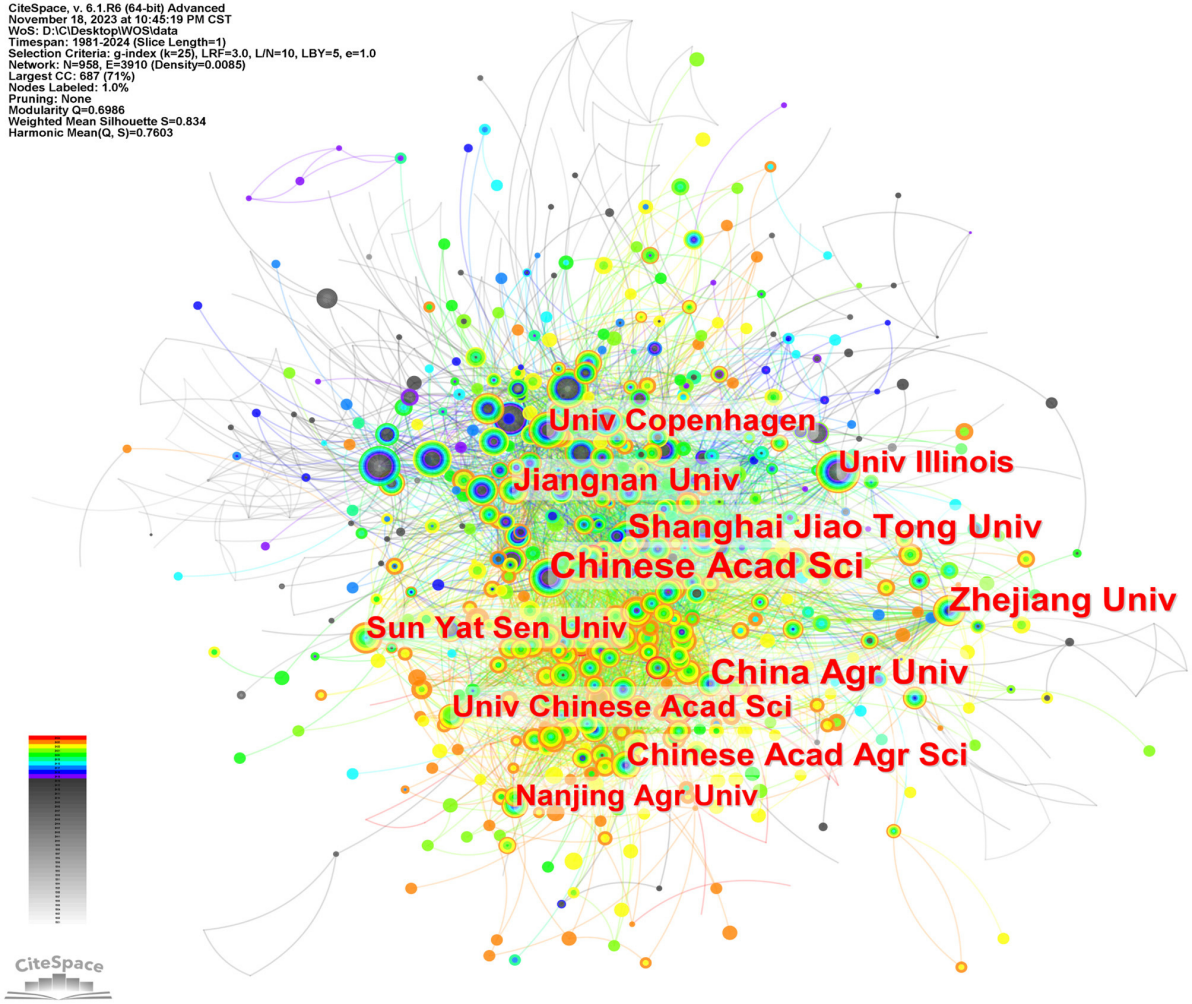
Cooperation networks between institutions via CiteSpace.

Figure 5 is a map of the institution's cooperation network. As shown in Table 1, Chinese Acad Sci (*n* = 331) published the most papers, followed by China Agr Univ (*n* = 252), Zhejiang Univ (*n* = 236), Shanghai Jiao Tong Univ (*n* = 196), Chinese Acad Agr Sci (*n* = 190), et al. Chinese Acad Sci (0.09) has a high centrality, indicating its importance as a link of institutional cooperation (Song et al., 2022).

**Table 1:** The top 10 institutes related to intestinal microbiota and lipids.

**Rank**	**Freq**	**Centrality**	**Label**
1	331	0.09	Chinese Acad Sci
2	252	0.03	China Agr Univ
3	236	0.05	Zhejiang Univ
4	196	0.04	Shanghai Jiao Tong Univ
5	190	0.02	Chinese Acad Agr Sci
6	160	0.03	Jiangnan Univ
7	140	0.01	Univ Chinese Acad Sci
8	134	0.07	Univ Copenhagen
9	128	0.04	Sun Yat Sen Univ
10	118	0.05	Univ Illinois

### 3.3 Analysis of journals

**Figure 6:**
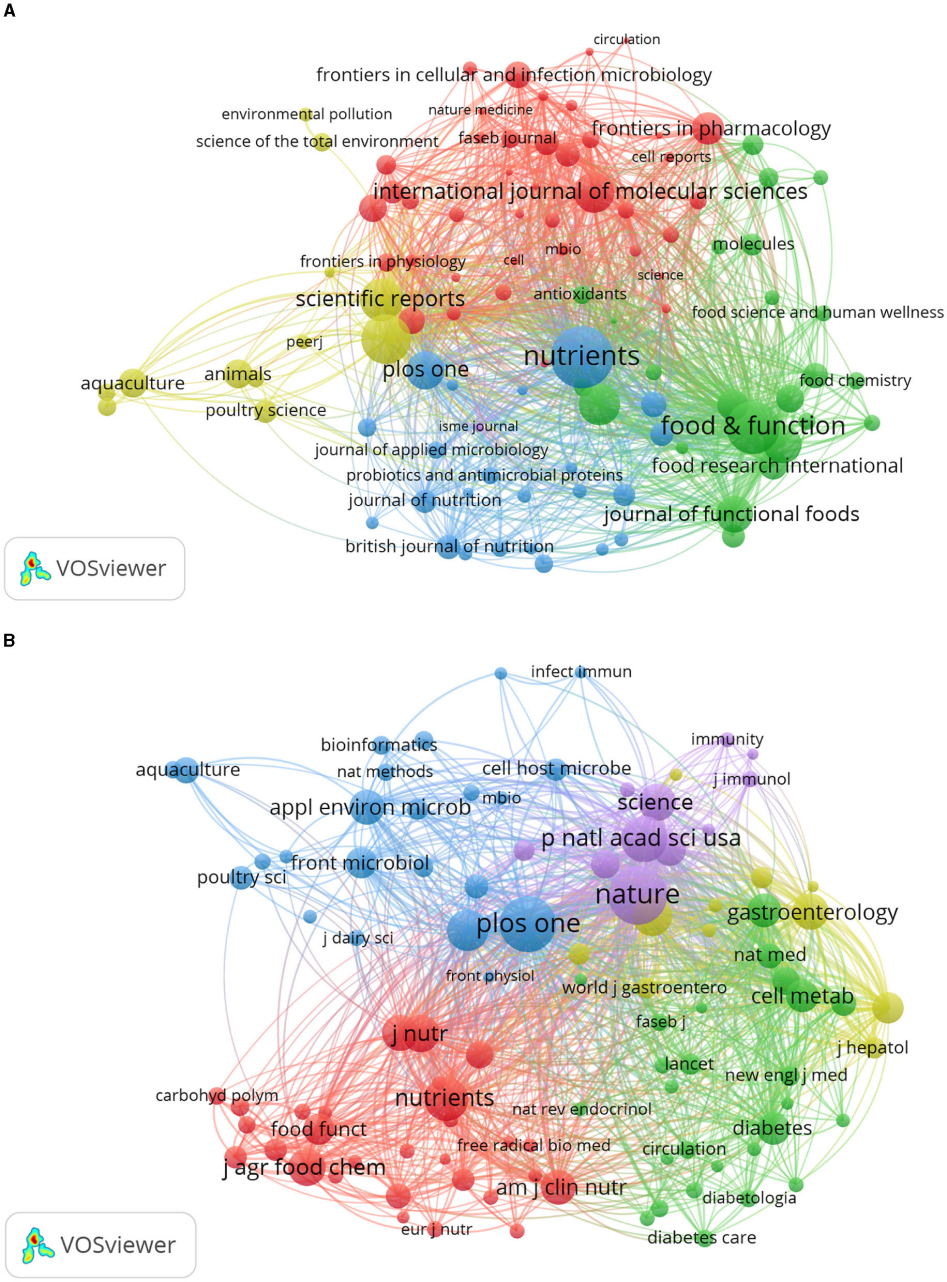
The network co-occurrence diagram of **(A)** journals and **(B)** co-cited journals.

VOSviewer was employed to identify journals with a high publication count and a significant co-citation frequency in this field. Figure 6 showcases the network co-occurrence diagram of journals (A) and co-cited journals (B). Nutrients emerged as the leading journal in gut microbiota and lipids, with a noteworthy publication count of 561 articles. Following closely behind were Food Funct (416), Front Microbiol (357), Sci Rep (265), Front Nutr (253), and others. Co-cited journals, on the other hand, denote those journals that multiple authors have cited. Through analysis, it was determined that Nature (18,181) secured the highest number of co-citations, trailed by PLoS One (17,053), Proc Natl Acad Sci U S A (12,705), Nutrients (11,543), Sci Rep (10,719), and others. Detailed information can be found in Table 2. The analysis of journals and co-cited journals allows for exploring preferred journals among authors and offers valuable references for future academic contributions.

**Table 2:** The top 10 journals and co-cited journals that published documents on intestinal microbiota and lipids.

**Ranking**	**Journal**	**Documents**	**Citations**	**IF/JCR**	**Co-cited journal**	**Citations**
1	Nutrients	561	14,893	5.9/Q1	Nature	18,181
2	Food Funct	416	9,239	6.1/Q1	PLoS One	17,053
3	Front Microbiol	357	7,265	5.2/Q1	Proc Natl Acad Sci U S A	12,705
4	Sci Rep	265	11,105	4.6/Q2	Nutrients	11,543
5	Front Nutr	253	1,892	5.0/Q1	Sci Rep	10,719
6	Int J Mol Sci	245	5,501	5.6/Q1	Gut	9,946
7	J Agric Food Chem	215	5,655	6.1/Q1	Science	9,165
8	PLoS One	215	12,370	3.7/Q2	J Nutr	9,025
9	J Funct Foods	211	4,149	5.6/Q2	J Agric Food Chem	8,983
10	Front Pharmacol	156	2,252	5.6/Q1	Neuron	8,724

**Figure 7:**
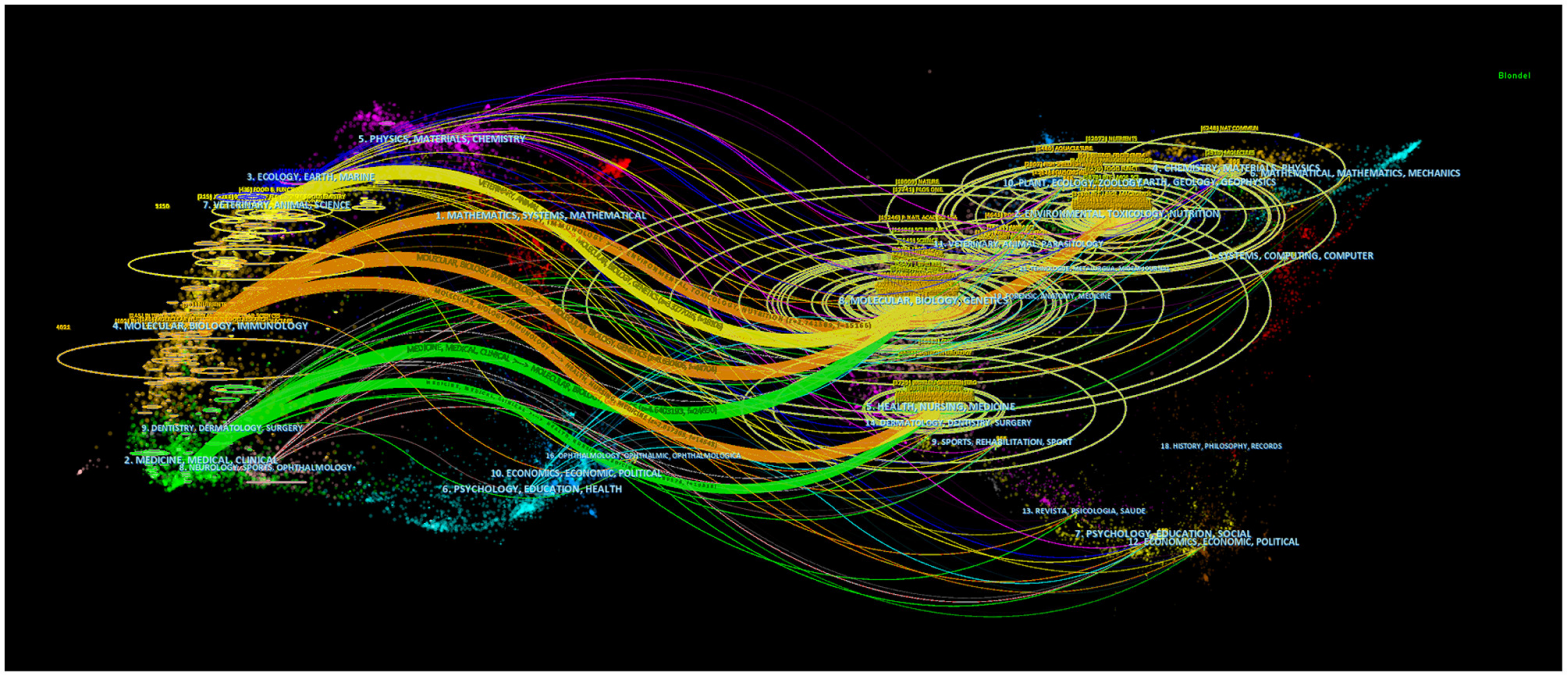
The dual-map overlay of journals related to intestinal microbiota and lipids.

Using dual-map overlay in journal analysis allows for the visualization of the cross-disciplinary distribution of journals, enabling the tracking of citation patterns and the evolution of scientific research centers (Yuan et al., 2023). In Figure 7, the left side depicts the distribution of journals within the cited literature. In contrast, the right side presents the distribution of journals in the citing literature, connected by color-coded paths. The analysis findings indicate that research on the correlation between intestinal flora and lipids predominantly intersects with fields such as veterinary and animal science, molecular biology, and immunology, as well as medicine, medical, and clinical domains. Scholars from disciplines like health, nursing, medicine, molecular biology, genetics, environmental toxicology, and nutrition frequently reference literature on intestinal flora and lipids.

### 3.4 Analysis of authors and co-cited authors

**Figure 8:**
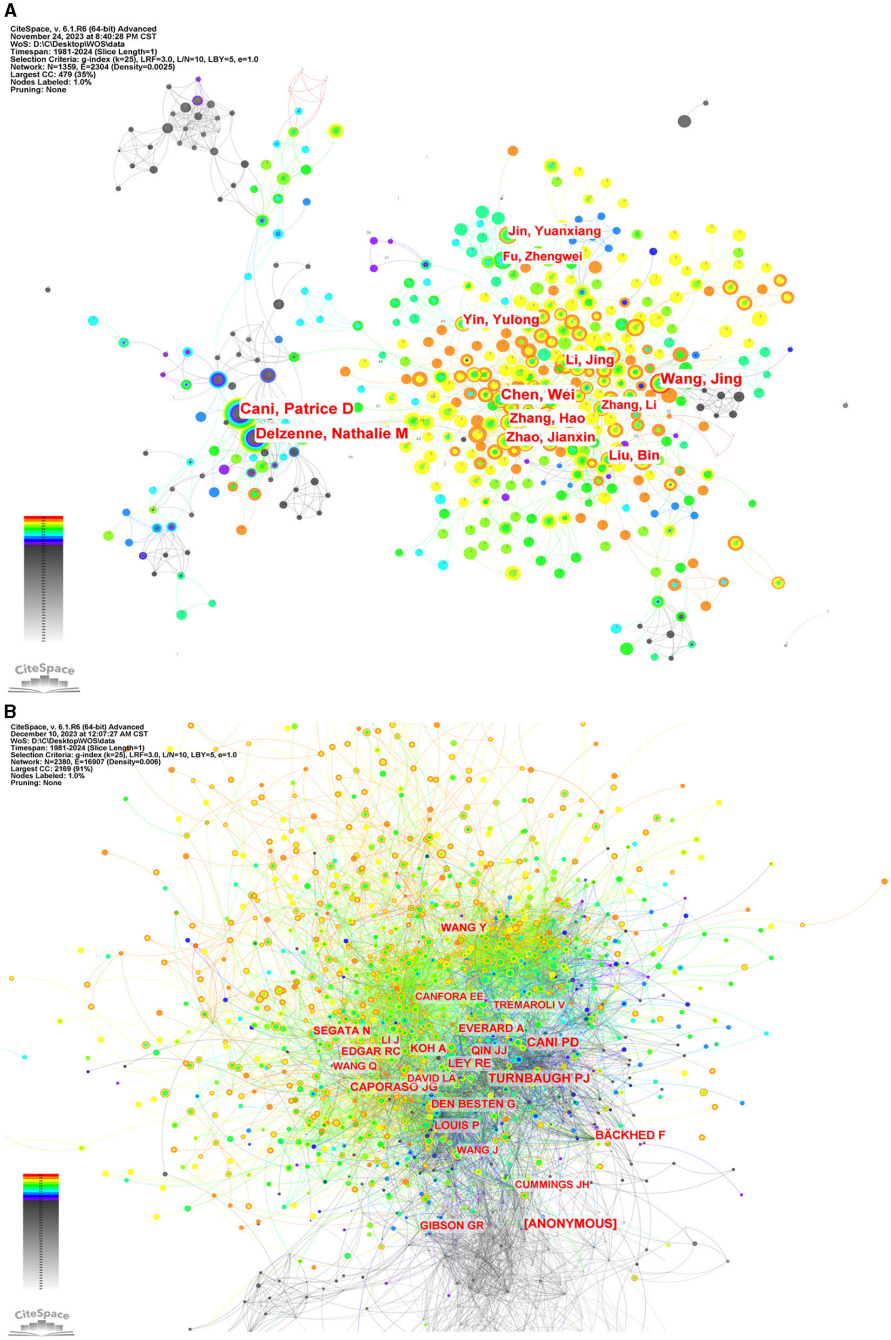
The network of authors via CiteSpace. **(A)** The network of authors in research of intestinal microbiota and lipids. **(B)** The network of cited authors in research of intestinal microbiota and lipids.

To ascertain the most influential authors in intestinal flora and lipids, an analysis of authors and co-cited authors was conducted using CiteSpace. Figure 8 illustrates the cooperative network diagram of authors (A) and co-cited authors (B), wherein each node represents an individual author. Furthermore, Table 3 enumerates the top ten authors with the most publications and co-citations. Notably, Patrice D Cani emerged as the most prolific author and frequently cited expert in this field. The top ten authors with the most number of documents were also Jing Wang (*n* = 68), Wei Chen (*n* = 64), Nathalie M Delzenne (*n* = 62), Jing Li (*n* = 48), and others. Nathalie M Delzenne had a high centrality (*n* = 0.07), representing his influence and importance in the field. The top ten authors with the most co-citations were also Peter J Turnbaugh (*n* = 2,015), [ANONYMOUS] (*n* = 2,007), Ruth E Ley (*n* = 1,685), Fredrik Bäckhed (*n* = 1,405), and others. The co-cited author with the highest centrality (*n* = 0.07) was anonymous.

**Table 3:** The top 10 authors with the most publications and citations.

**Ranking**	**Author**	**Frequency**	**Centrality**	**Cited author**	**Frequency**	**Centrality**
1	Patrice D Cani	82	0.01	Patrice D Cani	2,198	0.06
2	Jing Wang	68	0.02	Peter J Turnbaugh	2,015	0.06
3	Wei Chen	64	0.03	[ANONYMOUS]	2,007	0.07
4	Nathalie M Delzenne	62	0.07	Ruth E Ley	1,685	0.02
5	Jing Li	48	0.03	Fredrik Bäckhed	1,405	0.02
6	Bin Liu	45	0.01	J Gregory Caporaso	1,183	0.01
7	Hao Zhang	44	0.01	Amandine Everard	1,065	0.02
8	Yulong Yin	43	0.01	Jiang-Jiang Qin	1,027	0.02
9	Jianxin Zhao	43	0	Robert C Edgar	968	0.01
10	Yuanxiang Jin	36	0	Gijs den Besten	829	0.01

### 3.5 Analysis of co-cited reference

**Figure 9:**
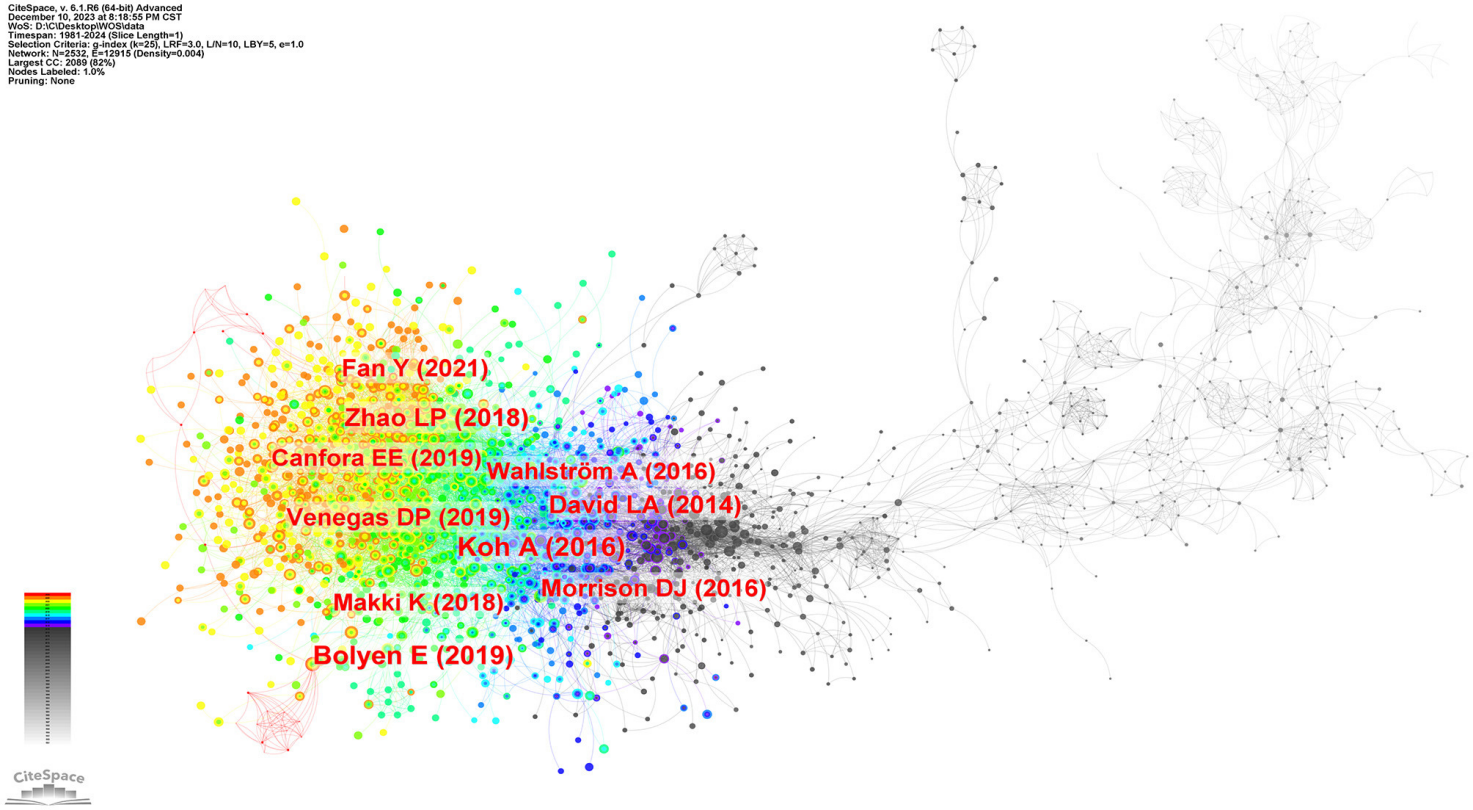
The network of co-cited reference on intestinal microbiota and lipids.

The research frontiers are explored by analyzing co-cited references (Qin et al., 2022). We selected a time frame from 1981 to 2024, divided it into annual intervals, and generated a co-cited literature network diagram (Figure 9) using the default system parameters. Table 4 presents data on the ten most frequently co-cited articles.

**Table 4:** The top 10 references with the most cited related to intestinal microbiota and lipids.

**Ranking**	**Frequency**	**Centrality**	**Cited reference**	**References**
1	393	0.01	From Dietary Fiber to Host Physiology: Short-Chain Fatty Acids as Key Bacterial Metabolites	Koh et al. (2016)
2	373	0.02	Reproducible, interactive, scalable and extensible microbiome data science using QIIME 2	Bolyen et al. (2019)
3	276	0.01	Gut bacteria selectively promoted by dietary fibers alleviate type 2 diabetes	Zhao et al. (2018)
4	251	0.01	Diet rapidly and reproducibly alters the human gut microbiome	David et al. (2014)
5	240	0.01	Short Chain Fatty Acids (SCFAs)-Mediated Gut Epithelial and Immune Regulation and Its Relevance for Inflammatory Bowel Diseases	Parada Venegas et al. (2019)
6	225	0	Gut microbiota in human metabolic health and disease	Fan and Pedersen (2021)
7	218	0	Formation of short chain fatty acids by the gut microbiota and their impact on human metabolism	Morrison and Preston (2016)
8	210	0	The Impact of Dietary Fiber on Gut Microbiota in Host Health and Disease	Makki et al. (2018)
9	207	0.02	Intestinal Crosstalk between Bile Acids and Microbiota and Its Impact on Host Metabolism	Wahlström et al. (2016)
10	198	0	Gut microbial metabolites in obesity, NAFLD and T2DM	Canfora et al. (2019)

The most frequently co-cited article (393) came from Koh et al. (2016) in intestinal flora and major lipids. The authors conducted a comprehensive review on the multifaceted role of short-chain fatty acids (SCFAs) in various physiological functions of the host. SCFAs are primarily produced through microbial fermentation of dietary fiber within the intestinal tract, and they actively participate in regulating the metabolism of both healthy and diseased individuals. Notably, SCFAs are also implicated in modulating host immune responses, such as the ability of butyric acid to stimulate the production of regulatory T cells (Tregs) and interleukin-10 (IL-10). As the authors of the second most frequently co-cited article (373), Bolyen et al. (2019) have developed a robust bioinformatic platform system called QIIMR2, designed explicitly for analyzing microbiome data. This innovative tool enhances our comprehension of the intricate microbial realm and further advances microbiome research. Subsequently, an article authored by Zhao et al. (2018) emerged, displaying a focus on randomized clinical trials and genomics methodologies. Their research unveiled compelling evidence that targeted manipulation of the gut microbiota capable of producing short-chain fatty acids may offer promising therapeutic avenues for addressing type 2 diabetes. These findings contribute novel insights into the treatment of this complex metabolic disorder.

Moreover, the co-cited literature possessing the highest centrality (0.18) is extracted, highlighting its profound significance and extensive influence within the academic community. In a series of mouse experiments conducted by Ley et al. (2005), it was discovered that obesity substantially impacts the diversity of gut microbiota. This finding indicates that targeted interventions aimed at modulating the community structure of the microbiota hold promise in regulating energy homeostasis among individuals affected by obesity.

**Figure 10:**
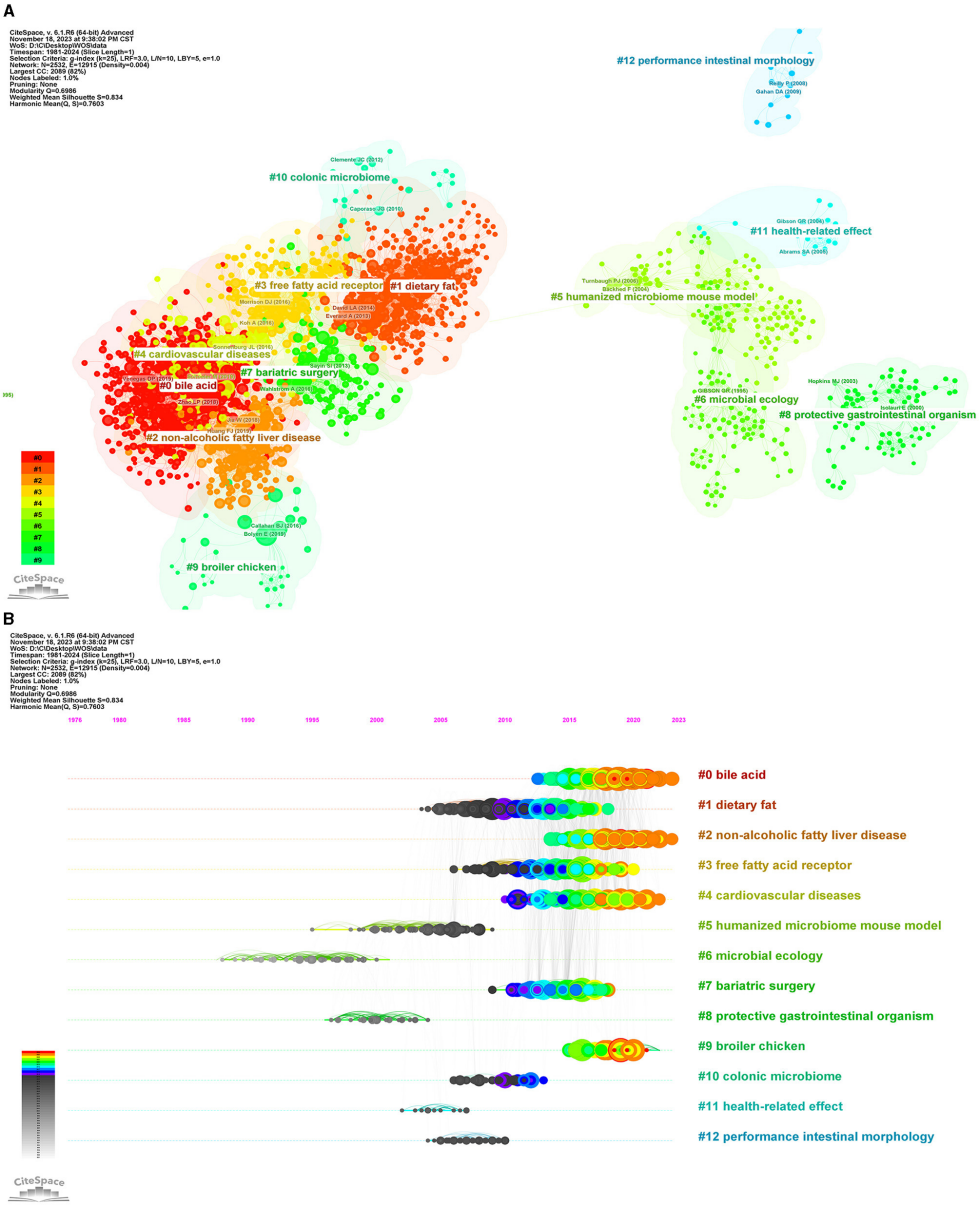
Analysis of co-cited reference. **(A)** The network of co-cited reference clusters. **(B)** The network of co-cited reference clustering timeline. Each color represents a cluster.

We used CiteSpace to analyze the co-cited references and obtained a cluster with significant cluster structure and high credibility (Q = 0.6986, S = 0.834). As shown in Figure 10, the cluster network diagram and the cluster time diagram of the co-cited literature were shown, respectively. Thirteen clusters were identified, categorized based on their respective cluster sizes: #0 bile acid, #1 dietary fat, #2 non-alcoholic fatty liver disease, #3 free fatty acid receptor, #4 cardiovascular diseases, #5 humanized microbiome mouse model, #6 microbial ecology, #7 bariatric surgery, #8 protective gastrointestinal organisms, #9 broiler chicken, #10 colonic microbiome, #11 health-related effects, and #12 performance intestinal morphology. Currently, the still active clusters were #0 bile acid, #2 non-alcoholic fatty liver disease, #4 cardiovascular diseases, and #9 broiler chicken. This classification system holds significant implications for forecasting research hotspots and anticipating emerging trends within intestinal flora and lipids.

**Figure 11:**
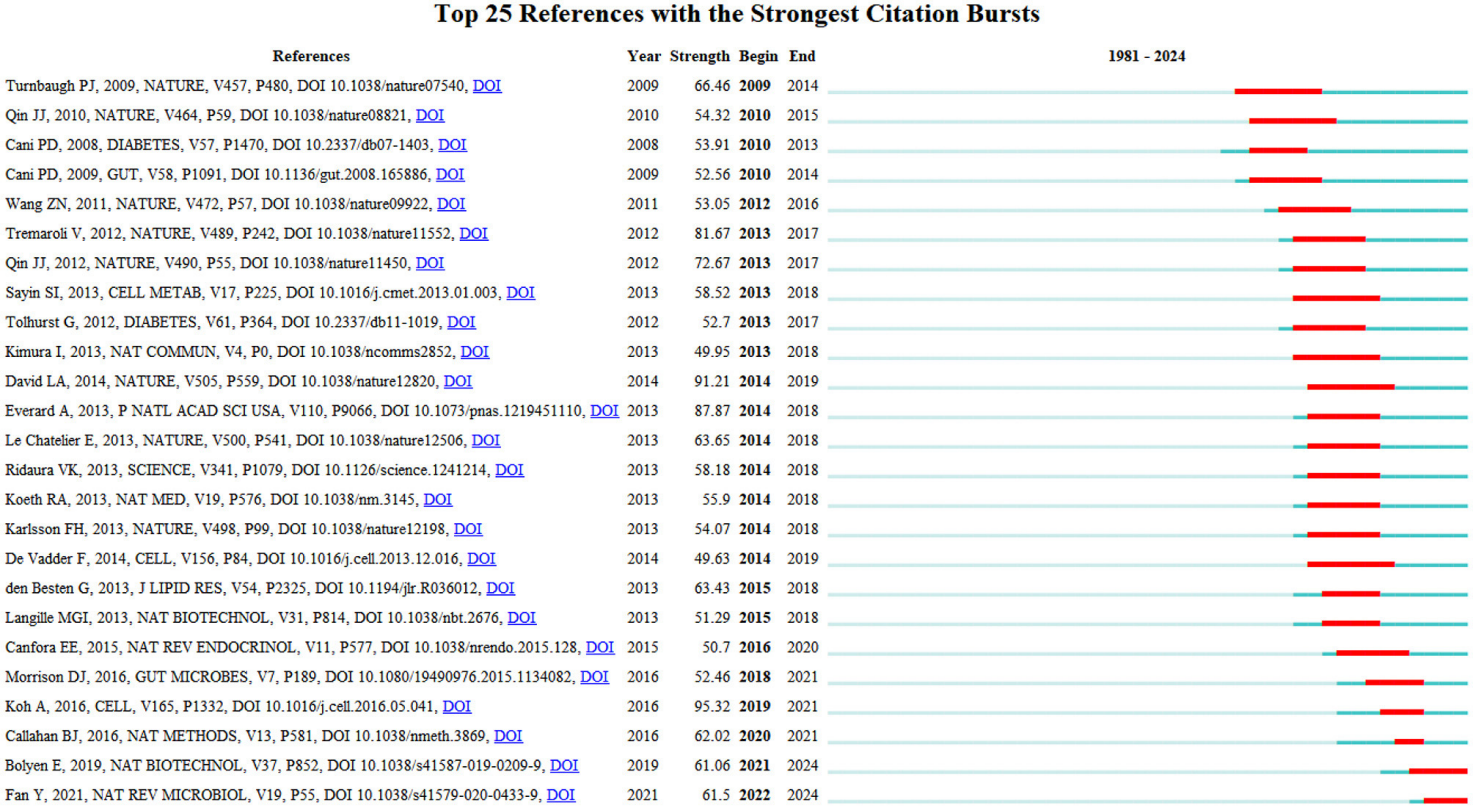
The top 25 references with the strongest citation bursts.

Figure 11 depicts the top 25 references with this field's most robust citation bursts. Notably, six publications have garnered significant attention from scholars over the past 6-year period, highlighting the prevailing research interests and emerging trends within this domain. These influential works indicate the current hotspots and avenues of investigation pursued by researchers in this field (Wang S. et al., 2023). The articles with the strongest citation bursts are Bolyen et al. (2019) and Fan and Pedersen (2021).

### 3.6 Analysis of keywords

**Figure 12:**
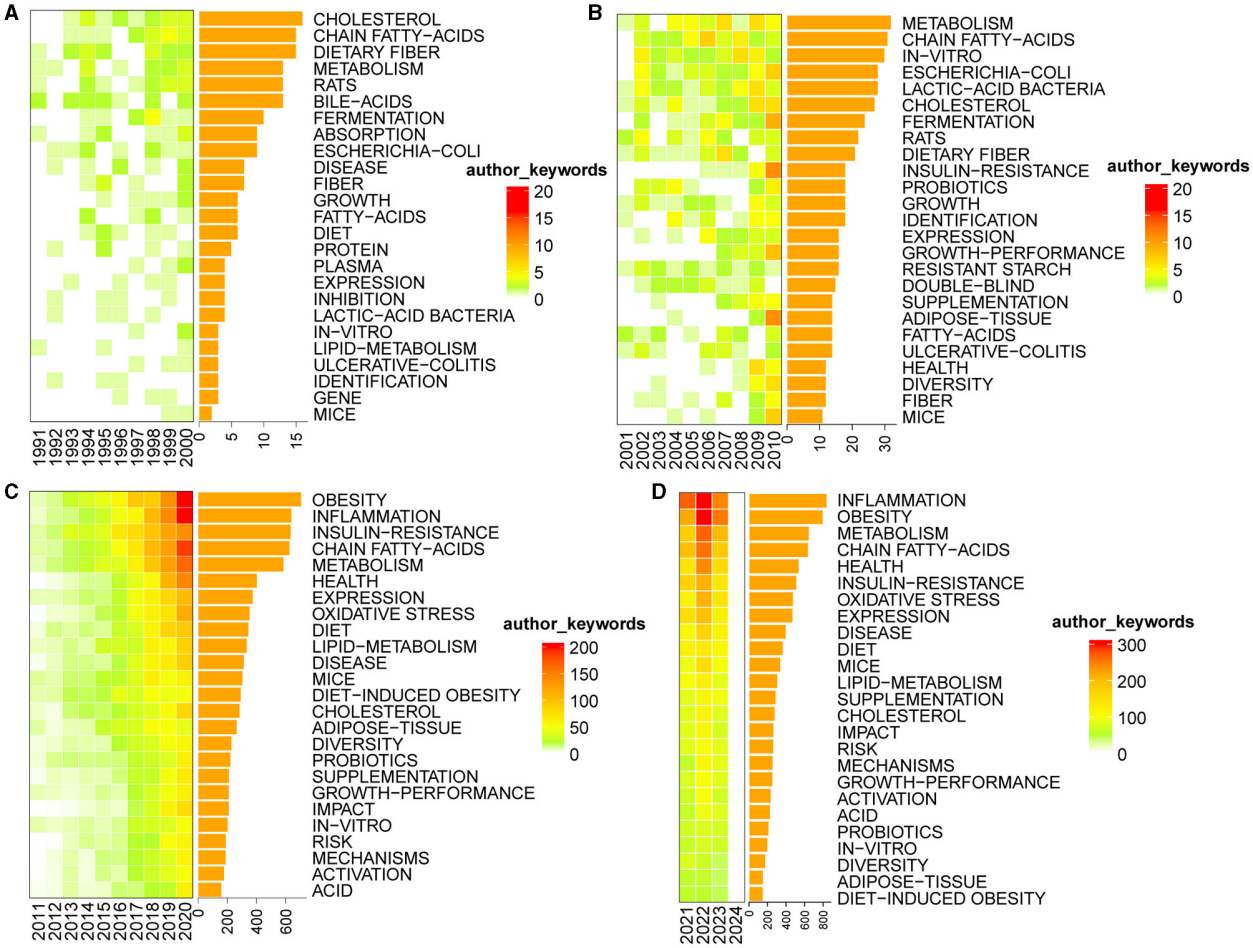
Heat maps of the top 25 most frequent keywords. **(A)** 1991–2000; **(B)** 2001–2010; **(C)** 2011–2020; **(D)** 2021–2024.

Keywords play a pivotal role in briefly summarizing the fundamental content of academic papers. Analyzing the frequency of keywords provides valuable insights into the dynamic nature of research content within a specific field of study (Wang J. W. et al., 2023). Based on the author's keyword analysis of the compiled dataset, we identified the top 25 keywords with the highest frequency. Subsequently, we constructed Figure 12, delineating the distribution of these keywords across different time periods, namely 1991–2000 (A), 2001–2010 (B), 2011–2020 (C), and 2021–2024 (D). This representation visually explores the temporal evolution of these prominent keywords and provides insights into the shifting research landscape within the specified timeframe. The intensity of color within each box reflects the relative frequency of the corresponding keyword occurrence in a given year. Darker hues indicate higher frequencies of the keyword in that specific year. Furthermore, the accompanying bar chart provides a quantitative representation of the frequency distribution for each keyword. We excluded four keywords: microbiota, intestinal microbiota, gut microbiota, and bacteria.

From 1991 to 2000, the keywords that exhibited the highest frequency were cholesterol, chain fatty-acids, and dietary fiber. From 2001 to 2010, keywords such as metabolism, chain fatty-acids, and *in-vitro* prominently emerged. Between 2011 and 2020, obesity, inflammation, insulin-resistance, chain fatty-acids, and metabolism stood out as the most frequently recurring keywords. As for the current timeframe of 2021 to 2024, the keywords with the highest frequency include inflammation, obesity, metabolism, and chain fatty-acids.

**Figure 13:**
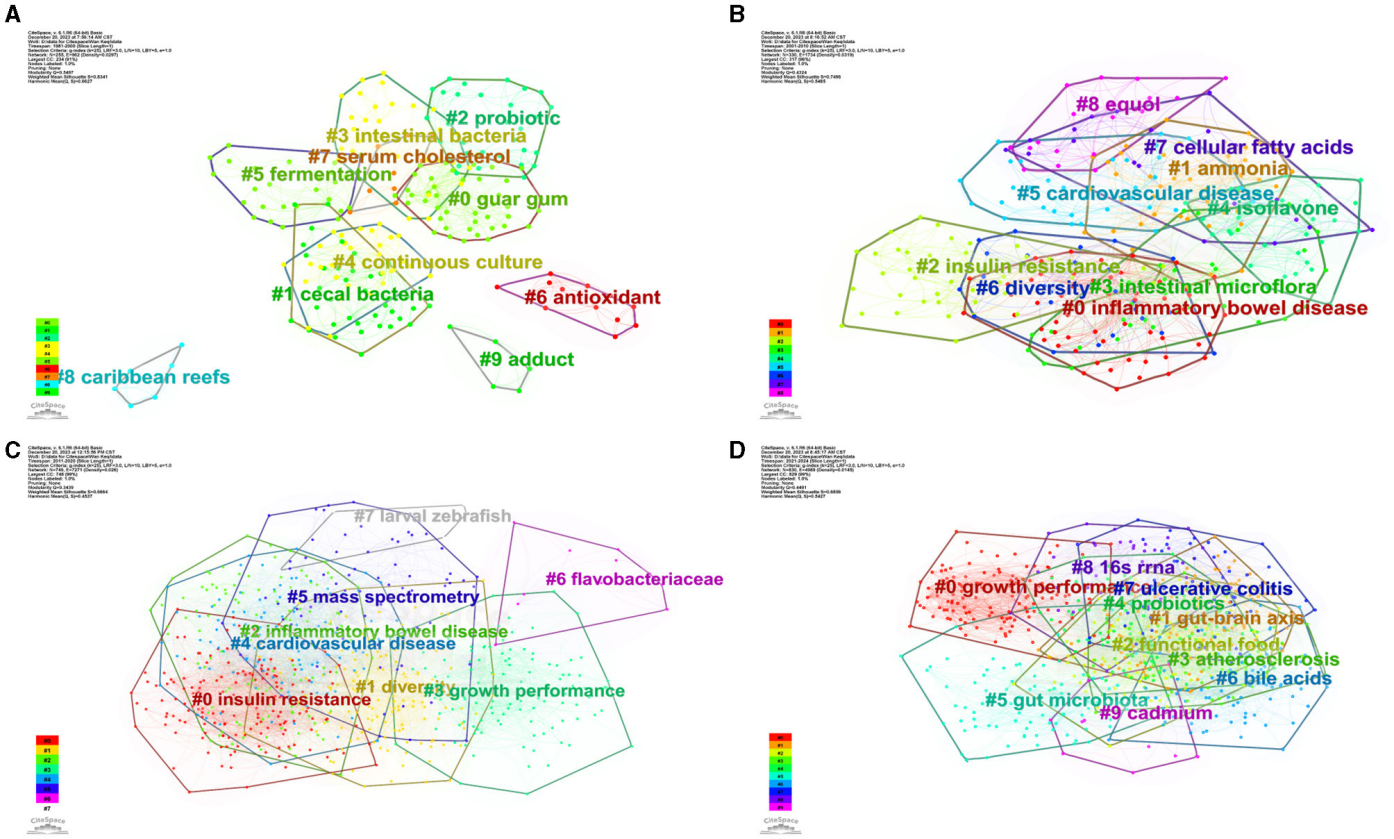
The network of keyword cluster analysis. **(A)** 1981–2000; **(B)** 2001–2010; **(C)** 2011–2020; **(D)** 2021–2024.

We conducted cluster analysis via CiteSpace, employing keywords from four distinct periods: 1981–2000 (A), 2001–2010 (B), 2011–2020 (C), and 2021–2024 (D), as shown in Figure 13. The resulting networks exhibited satisfactory modular scores (Q > 0.3) and significant contour scores (S > 0.6), indicating the presence of well-defined clusters within the analyzed data (Amrapala et al., 2023).

In Figure 13A, the most crucial cluster was “guar gum,” followed by “cecal bacteria,” “probiotic,” “intestinal bacteria,” “continuous culture,” “fermentation,” and “antioxidant.” In Figure 13B, the most important cluster was “inflammatory bowel disease,” followed by “ammonia,” “insulin resistance,” “intestinal microflora,” “isoflavone,” “cardiovascular disease,” “diversity,” “cellular fatty acids,” and “equol.” In Figure 13C, the most important cluster was “insulin resistance,” followed by “diversity,” “inflammatory bowel,” “growth performance,” “cardiovascular disease,” “mass spectrometry,” “flavobacteriaceae,” and “larval zebrafish.” In Figure 13D, the most important cluster was “growth performance,” followed by “gut-brain axis,” “functional food,” “atherosclerosis,” “probiotics,” “gut microbiota,” “bile acids,” “ulcerative colitis,” “16s rrna,” and “cadmium.” Additional details on the clusters can be found in the Supplementary material.

## 4 Discussion

### 4.1 Summary of main findings

This study represents the first bibliometric analysis examining the relationship between gut microbiota and major lipids. Various bibliometric tools, including CiteSpace, VOSviewer, R software, and Scimago Graphica, were employed to comprehensively analyze critical indicators such as country, institutions, journals, authors, keywords, and referenced literature within this research domain.

There has been an upward trend in published papers focusing on intestinal flora and major lipid fields. The annual number of published papers surpassed 1,000 after 2019 and reached its pinnacle in 2022. These findings suggest an increasing level of interest and contributions from scholars in this particular field. China emerged as the leading contributor in terms of publications in the realm of intestinal flora and major lipid fields. The Chinese Acad Sci stood out as the institution with the most published papers in this field, further highlighting China's prominence. Food Func published the most articles on intestinal flora and lipids among the various journals. At the same time, Nature assumed a crucial role as the most co-cited journal, serving as a valuable reference for scholars' contributions. Additionally, Patrice D Cani garnered notable distinction as this domain's prolific and most frequently co-cited author.

Several active clusters emerged through the research's co-cited reference clustering network, including #0 bile acid, #2 non-alcoholic fatty liver disease, #4 cardiovascular diseases, and #9 broiler chicken. We conducted a comprehensive analysis utilizing frequency and cluster analysis techniques to examine keyword trends across four distinct time periods: 1981–2000, 2001–2010, 2011–2020, and 2021–2024. Based on our analyses, four prominent research trends have emerged from the network analysis on gut microbiota and major lipids: (1) biochemical pathways, (2) exploration of diseases, (3) intervention and effect, and (4) health and diet.

### 4.2 Biochemical pathways

Biochemical pathways include metabolic pathways and biochemical compounds associated with gut flora and lipids. Our analysis has identified a range of pertinent factors falling within this category, including bile acid, chain fatty-acids, oxidative stress, lipid metabolism, mechanisms, expression, free fatty acid receptors, ammonia, and the gut-brain axis.

Extensive research has demonstrated the regulatory role of gut microbes in the metabolism of carbohydrates, lipids, and amino acids, thereby significantly influencing human health, and metabolic diseases (Wang and Yu, 2020). Intestinal flora actively participates in the degradation and absorption of nutrients (Moszak and Szulińska, 2020), while also generating a wide array of metabolites via diverse metabolic pathways. The extensively researched metabolites encompass short-chain fatty acids, bile acids, tryptophan, and its derivatives. Short-chain fatty acids are the end products resulting from intestinal microbiota-mediated fermentation of dietary fiber (Den Besten et al., 2013). These short-chain fatty acids contribute to the promotion of intestinal barrier integrity, regulation of glucose and lipid metabolism, and modulation of inflammation, blood pressure, and immune responses (Nogal et al., 2021). The interplay between host-derived enzymes and those derived from intestinal microbiota substantially influences the composition of the bile acid pool (Collins et al., 2023). The diversity of bile acids within this pool can be attributed to the varying types of intestinal bacteria (Mancin et al., 2023). Bile acids exert their regulatory effects on host metabolism and function by activating distinct bile acid receptors, which subsequently modulate signal transduction pathways (Cai et al., 2022). In the gastrointestinal tract, the microbiome exerts direct or indirect control over the three primary tryptophan metabolic pathways, leading to the formation of 5-hydroxytryptamine (5-HT) (Yano et al., 2015), kynuridine (Kyn) (Clarke et al., 2013), and indole derivatives (Zelante et al., 2013). These metabolites, including tryptophan, indole, and kynuridine, play crucial roles in modulating neuroendocrine and intestinal immune responses (Gao et al., 2018, 2020). Mechanistic relationships between lipid metabolism and microbial metabolites have been identified, encompassing the intricate biosynthesis processes and lipids' degradation, including fatty acids, triglycerides, and cholesterol (Wang et al., 2016). Perturbation in lipid metabolism has been implicated in the pathogenesis of various metabolic disorders such as obesity, atherosclerosis, and non-alcoholic liver disease (Schoeler and Caesar, 2019). These insights illuminate the vital role of gut microbiota in modulating lipid metabolism and its implications in the development and progression of metabolic diseases.

In recent years, the gut-brain axis has emerged as a noteworthy cluster, establishing a vital connection between intestinal flora and the central nervous system. Specific lipids, notably short-chain fatty acids, have been identified as regulators of both peripheral and central pathological processes, potentially contributing to developing and progressing central nervous system-related diseases (Russo et al., 2018). Moreover, it has been established that tryptophan plays a pivotal role in the microbial-gut-brain axis (Roth et al., 2021). Tryptophan and its metabolites contribute to the maturation and maintenance of both the central and enteric nervous systems, thus offering a promising avenue for the treatment of central nervous system disorders and psychopathological conditions (Kennedy et al., 2017). Nevertheless, further investigation by scholars is warranted to fully explore and comprehend this potential target. These findings underscore the intricate interplay between gut microbiota, lipids, and the central nervous system, highlighting the significance of exploring this axis in understanding and managing neurological disorders.

### 4.3 Exploration of diseases

Our keywords and co-cited literature analysis revealed a notable shift in scholarly research on intestinal flora and lipids toward clinical applications, focusing on diseases characterized by dysregulated lipid metabolism. Inflammatory bowel disease, cardiovascular disease, insulin resistance, atherosclerosis, ulcerative colitis, non-alcoholic fatty liver disease, and obesity emerged as the most prevalent keywords and clusters in this field. These findings indicated the substantial attention researchers gave to investigating the interplay between intestinal flora, lipids, and the pathogenesis of these clinically relevant disorders.

Dysregulated lipid metabolism is implicated in the pathogenesis of numerous diseases, including but not limited to obesity (Li Y. et al., 2020; Aron-Wisnewsky et al., 2021), insulin resistance (Duttaroy, 2021), inflammatory bowel disease (Brown and Walker, 2016), atherosclerosis (Poznyak et al., 2020), ulcerative colitis (Ma et al., 2024), cardiovascular disease (Poznyak et al., 2020), and non-alcoholic fatty liver disease (Ipsen et al., 2018). Mounting evidence has established a significant association between lipid metabolism and intestinal microbiota (Yin et al., 2018; Wang Y. et al., 2023). However, the question of whether these diseases are all linked to gut microbiota remains a subject of debate, and further evidence is required to establish a definitive connection. Significantly, lipid metabolism disorders often coincide and exhibit a close interrelation (Deprince et al., 2020). For instance, hyperlipidemia has a propensity to expedite the progression of atherosclerosis (Agrawal et al., 2018), while the presence of diabetes enhances the likelihood of cardiovascular diseases (Furse, 2022). Given these circumstances, researchers must delve deeper into the multifaceted pathogenesis of lipid metabolism disorders to understand these diseases comprehensively. Furthermore, it is important to note that these diseases can be multifactorial in nature, with factors like immune-mediated processes influencing the onset and progression of conditions such as ulcerative colitis (Glassner et al., 2020). Consequently, there is a pressing need for extensive investigations to unravel the intricate relationship between these diseases and the gut microbiota, and to use the gut microbiota for treatment.

In recent years, the association between neurodegenerative diseases and intestinal flora has garnered significant attention among researchers. Studies have indicated that individuals with Parkinson's disease exhibit reduced levels of bacteria that produce short-chain fatty acids, resulting in increased intestinal permeability and exposure of the intestinal nerve plexus to toxins. This exposure subsequently leads to abnormal aggregation of alpha-synuclein fibrae (Vascellari et al., 2020; Hirayama and Ohno, 2021). Additionally, experiments conducted on Alzheimer's disease mice have demonstrated that modulation of gut microbiota through probiotic intervention can induce changes in lipid composition, thereby exerting a pivotal role in insulin sensitivity regulation and inflammation control (Bonfili et al., 2022). These significant findings offer novel insights into potential therapeutic approaches for neurodegenerative diseases.

### 4.4 Intervention and effect

In this section, we elucidated the principal interventions and effects delineated by scholars investigating the field of gut microbiota and lipids. From the clusters of keywords, it could be observed that guar gum, probiotics, isoflavone, growth performance, broiler chicken, and cadmium all fall under this study category.

Several studies have revealed the potential therapeutic significance of guar gum in managing metabolic syndrome, specifically in the treatment of insulin resistance (Landin et al., 1992), hypercholesterolemia (Frias and Sgarbieri, 1998), lipid-lowering (Khan et al., 1981), obesity (Li et al., 2023), and other lipid metabolism disorders. However, further research and exploration are warranted to delve deeper into these therapeutic effects. Probiotics have demonstrated the ability to enhance blood sugar and lipid metabolism in individuals with diabetes and mitigate inflammation and oxidative stress (Stojanov and Berlec, 2020; Dai et al., 2022). Consequently, they hold significant potential as a therapeutic approach to managing these interconnected diseases. Isoflavones have great potential to improve quality of life by lowering cholesterol, lowering blood lipids, and enhancing lipid metabolism (Kim, 2021; Huang et al., 2022). Furthermore, isoflavones exhibit the capacity to modulate inflammatory signaling pathways, impact intestinal barrier function, and influence microbial flora (Wu et al., 2020). Isoflavones possess a structural resemblance to estrogen and can also exert their effects via estrogen receptors (Ghimire et al., 2022). Its specific mechanism and clinical application are still being studied and explored. Growth performance is mainly reflected in animal husbandry through the correlation between intestinal flora and lipids, which improves biological quality (Silva-Guillen et al., 2020). Exposure to cadmium presents a considerable health risk, as it perturbs lipid metabolism and contributes to the development of atherosclerosis (Li X. et al., 2020). Concurrently, hepatic accumulation of cadmium is responsible for inducing liver damage (Liu et al., 2022). The use of prebiotics represents a potential strategy for mitigating cadmium toxicity (Tinkov et al., 2018). This discovery unveils novel therapeutic targets and insights for treating cadmium-induced disorders, necessitating further investigation into the underlying mechanisms (Lin C. Y. et al., 2023).

Given the association between gut microbiota and disease pathogenesis, fecal microbiota transplantation (FMT) emerges as a viable therapeutic strategy (Vindigni and Surawicz, 2017). It has demonstrated efficacy in treating recurrent Clostridium difficile infections, highlighting its potential as a treatment modality (Cheng and Fischer, 2023). Nevertheless, the specific application of FMT in other diseases warrants further investigation and consideration (Porcari et al., 2023), with the anticipation of novel advancements in this field.

### 4.5 Health and diet

In our analysis, we also identified a research direction on health and diet, “functional food,” “bariatric surgery,” “health-related effect,” “health,” “diet,” “diet-induced obesity,” “dietary fiber,” and others, all of which fell under this category. This extensive attention from scholars proves the significance of this research direction.

To prevent and intervene in diseases associated with gut microbiota and lipids, scholars have employed functional foods, such as dietary fiber, for their potential contributions to human health (Prosky, 2000). Diminished consumption of dietary fiber can have detrimental effects on the composition of the gut microbiota (Fu et al., 2022). Recent studies have revealed that a low-fiber diet can induce cognitive impairment by perturbing the gut microbiota-hippocampal axis (Shi et al., 2021), offering significant research implications for the development of degenerative diseases. The intricate interplay between dietary fiber and intestinal flora may present novel therapeutic avenues and insights for inflammatory bowel disease (Rasmussen and Hamaker, 2017), type 2 diabetes (Kovatcheva-Datchary et al., 2015). Developing and identifying functional foods that promote healthier eating habits is paramount in preventing and intervening in related diseases. Encouraging more researchers to contribute to this field of study is imperative. Obesity, characterized as a chronic lipid metabolic disorder, poses a significant health risk and is often associated with numerous complications. Bariatric surgery has been extensively investigated as an efficacious treatment modality, with ongoing discussions regarding its association with the gut microbiota (Gasmi et al., 2023). Additionally, exploring the potential of genetic modification in obese patients holds promise, leveraging the functional interconnections between the intestinal microbiota and lipid metabolism (Ciobârca et al., 2020).

### 4.6 Research trends and future directions

Visual analysis of co-cited references and keywords can predict the research trend and future direction in the field of intestinal microbiota and lipids (Lin X. et al., 2023). In addition to the abovementioned research that continues to warrant scholarly attention and investigation, we have also identified the utilization of 16S rRNA sequencing as a sequencing technology. This method enables the elucidation of microbial composition, exploration of microbial diversity, identification of potential pathogens, and monitoring of therapeutic effects, and it offers guidance for personalized nutritional interventions (Zhang et al., 2021).

In conclusion, the relationship between gut microbiota and lipids has been partially elucidated, exerting a profound impact on the initiation and progression of diverse metabolic disorders. Scholars have investigated the factors that influence and perturb the metabolism of gut microbiota and lipids and have explored interventions utilizing functional foods and other therapeutic modalities to mitigate and prevent such diseases, thereby enhancing human health. This field of study is of utmost importance and merits further exploration by future researchers. We will continue to monitor advancements in this domain closely.

### 4.7 Strengths and limitations

As the inaugural metrical analysis of literature within the gut microbiota and lipids field, our study provides a comprehensive assessment of the pertinent knowledge, offering a scientific foundation and future research direction in this realm. We harnessed several bibliometric tools, leveraging their strengths to complete our analysis. Nevertheless, this study possesses certain limitations. Firstly, our inclusion criteria solely considered English articles, possibly overlooking contributions from non-English publications within this field. Additionally, our analysis focused exclusively on major lipids, potentially neglecting the significance of minor lipids, which may also exert a notable impact. Consequently, we intend to prioritize addressing these concerns in subsequent research endeavors.

## 5 Conclusions

A comprehensive quantitative analysis was conducted on a dataset comprising 12,693 articles on gut microbiota and major lipids obtained from the Web of Science Core Collection (WoSCC). This analysis revealed several key findings:

1. The number of publications in this field exhibited a steady upward trend, with interdisciplinary collaboration being prominent. China emerged as the leading country regarding publication output, while the USA displayed the closest ties with other countries. Chinese Acad Sci, located in China, exhibited the highest publication activity within this domain.
2. Among the journals, Nutrients published the most significant articles, while Nature recorded the highest number of co-citations in this field. Patrice D Cani emerged as the most prolific author, both in terms of publications and co-citations.
3. Through an analysis of keywords and co-cited literature, four prominent research trends were identified: “biochemical pathways,” “exploration of diseases,” “intervention and effect,” and “health and diet.” These findings underscore the importance of further scholarly attention in this field. In the future, more scholars need to pay attention to this field and strive to explore human health.

## Data availability statement

The raw data supporting the conclusions of this article will be made available by the authors, without undue reservation.

## Author contributions

WS: Writing – review & editing, Writing – original draft, Project administration, Funding acquisition, Conceptualization. KW: Writing – review & editing, Writing – original draft, Software, Methodology, Formal analysis, Data curation. JG: Writing – review & editing, Writing – original draft, Visualization, Software, Formal analysis, Data curation. GJ: Writing – review & editing, Validation, Supervision, Resources, Project administration, Funding acquisition, Conceptualization. LS: Writing – review & editing, Validation, Supervision, Project administration, Investigation, Funding acquisition, Conceptualization.
