# Supplementary material for: Visual trends and hot research on the relationship between intestinal microbiota and major lipids: a bibliometric analysis
Source: Front Microbiol. 2024 Apr 30;15:1361439. doi: 10.3389/fmicb.2024.1361439 (PMC11091250; doi:10.3389/fmicb.2024.1361439)

***Supplementary Material***

**Supplementary Table 1** Cluster information of keywords network (1981-2000)

| Cluster ID | Size | Silhouette | Mean (year) | Cluster label | Top Terms (log-likelihood ratio, p-level) |
| --- | --- | --- | --- | --- | --- |
| 0 | 44 | 0.781 | 1994 | guar gum | guar gum (8.76, 0.005); metabolism (5.83, 0.05); fermentation (3.76, 0.1); rat (3.08, 0.1); clostridium strain (2.9, 0.1) |
| The top 20 most frequent keywords: bile acid, cholesterol, rat, metabolism, absorption, serum, assimilation, cell proliferation, excretion, acetate, lactobacilli, cellulose, invivo, deconjugation, fructo oligosaccharide, microflora, conventional rat, germ-free rat, guar gum, acidophilus. | | | | | |
| 1 | 37 | 0.793 | 1995 | cecal bacteria | cecal bacteria (7.51, 0.01); volatile fatty acid (4.02, 0.05); lipid peroxidation (4.02, 0.05); vibrios (3.74, 0.1); competitive exclusion bacteria (3.74, 0.1) |
| The top 20 most frequent keywords: intestinal microflora, colon, diet, fece, breast cancer, volatile fatty acid, cecal bacteria, germ free, brachionus plicatili, plant sterol, protection, broiler chicken, dietary lactose, low density lipoprotein, digestion, bifidobacterium, binding globulin, broiler chick, alginolyticus, 1 nitropyrene. | | | | | |
| 2 | 31 | 0.855 | 1995 | probiotic | probiotic (7.06, 0.01); clostridium difficile (7.06, 0.01); hypocholesterolemic (5.34, 0.05); human intestinal bacterium (5.34, 0.05); intestinal immune system (5.34, 0.05) |
| The top 20 most frequent keywords: fecal flora, disease, human fecal flora, lactobacillus acidophilus, human fece, bacteroides fragili, short chain fatty acid, plasma cholesterol level, bifidobacterium longum, intestinal microbiota, 1st 2 year, cholic acid, clostridium difficile, bacterial, bacterial colonization, continuous culture system, anti tumor activity, digoxin, dissimilatory sulfate reduction, colonisation resistance | | | | | |
| 3 | 31 | 0.846 | 1994 | intestinal bacteria | intestinal bacteria (9.76, 0.005); 7 alpha-dehydroxylation (4.86, 0.05); yogurt like product (4.86, 0.05); fermented milk (4.86, 0.05); epithelium (4.86, 0.05) |
| The top 20 most frequent keywords: escherichia coli, gastrointestinal tract, intestinal bacteria, diarrhea, fish oil, parenteral nutrition, critically ill, bacterial translocation, growing pig, beta galactosidase, anaerobe cultural count, d phosphogalactoside galactohydrolase, anti gal, 7 alpha-dehydroxylation, amino acid synthesis, antidiabetic activity, antibody, colon carcinogenesis, bacterial cholyltaurine hydrolase, amino acid requirement | | | | | |
| 4 | 31 | 0.799 | 1995 | continuous culture | continuous culture (6.48, 0.05); chain fatty acid (6.48, 0.05); colorectal cancer (6.48, 0.05); resistant starch (6.48, 0.05); fecal ph (3.23, 0.1) |
| The top 20 most frequent keywords: dietary fiber, chain fatty acid, short-chain fatty acid, fermentation, fatty acid, anaerobic bacteria, 16s ribosomal rna, antibiotic-associated diarrhoea, resistant starch, carcinogenesis, immune system, human colon, epithelial cell proliferation, healthy subject, large intestine, carbohydrate fermentation, colorectal cancer, blood lipid, protein, invitro | | | | | |
| 5 | 27 | 0.866 | 1994 | fermentation | fermentation (9.37, 0.005); breast milk (9.07, 0.005); oligosaccharides (9.07, 0.005); children (5.47, 0.05); intestinal flora (5.47, 0.05) |
| The top 20 most frequent keywords: bacteria, bifidobacteria, antibiotics, intestinal flora, degradation, coprostanol, colon cancer, breast milk, oligofructose, adaptation, branched-chain fatty acid, endogenous lipid, colonic flora, fecal fatty acid, ammonia, conversion, atlantic salmon, alterfunditum, aeromonas salmonicida, cholesterol absorption | | | | | |
| 6 | 13 | 0.909 | 1998 | antioxidant | antioxidant (12.22, 0.001); atherosclerosis (6.06, 0.05); 4-oxo-flavonoids (6.06, 0.05); f-2-isoprostanes (6.06, 0.05); quercetin (6.06, 0.05) |
| The most frequent keywords: soybean isoflavone, cardiovascular disease, in vitro, phyto estrogen, fecal sample, alpha tocopherol, antioxidant activity, plasma, ascites tumor cell, antitumoral effect, dependent protein kinase, cultured mammalian cell, 8 iso prostaglandin f2 alpha | | | | | |

**Supplementary Table 2** Cluster information of keywords network (2001-2010)

| Cluster ID | Size | Silhouette | Mean (year) | Cluster label | Top Terms (log-likelihood ratio, p-level) |
| --- | --- | --- | --- | --- | --- |
| 0 | 59 | 0.685 | 2003 | inflammatory bowel disease | inflammatory bowel disease (19.75, 1.0E-4); aberrant crypt foci (9.85, 0.005); bifidobacteria (7.99, 0.005); oligonucleotide probe (6.56, 0.05); galacto-oligosaccharides (6.56, 0.05) |
| Representative keywords: bacteria, chain fatty acid, in vitro, escherichia coli, gastrointestinal tract, inulin, inflammatory bowel disease, bifidobacteria, aberrant crypt foci, in situ hybridization | | | | | |
| 1 | 47 | 0.696 | 2006 | ammonia | ammonia (17.18, 1.0E-4); barley (17.18, 1.0E-4); pig (15.11, 0.001); fucoidan (12.87, 0.001); laminarin (12.87, 0.001) |
| Representative keywords: dietary fiber, fermentation, intestinal microbiota, growth performance, nutrient digestibility, resistant starch, fiber, fatty acid, concentration, serum cholesterol, fructooligosaccharide | | | | | |
| 2 | 42 | 0.743 | 2006 | insulin resistance | insulin resistance (29.24, 1.0E-4); adipose tissue (21.88, 1.0E-4); gut microbiota (20.31, 1.0E-4); obesity (16.4, 1.0E-4); necrosis factor alpha (14.56, 0.001) |
| Representative keywords: gut microbiota, rat, microflora, insulin resistance, intestinal flora, adipose tissue, lipid metabolism, necrosis factor alpha, diet, induced obesity, model | | | | | |
| 3 | 36 | 0.74 | 2004 | intestinal microflora | intestinal microflora (19.13, 1.0E-4); flora (15.35, 1.0E-4); double blind (10.66, 0.005); cytokine (7.66, 0.01); mineral density (7.66, 0.01) |
| Representative keywords: intestinal microflora, probiotics, flora, short-chain fatty acid, double blind, ulcerative coliti, mice, short chain fatty acid, butyrate, alpha | | | | | |
| 4 | 35 | 0.784 | 2005 | isoflavone | isoflavone (14.25, 0.001); assimilation (9.48, 0.005); plasma lipids (9.48, 0.005); fermented milk product (9.48, 0.005); yogurt (9.48, 0.005) |
| Representative keywords: lactic acid bacteria, cholesterol, lactobacillus acidophilus, serum, oligofructose, bile acid, fermented milk product, colon cancer, plasma lipid, propionate | | | | | |
| 5 | 34 | 0.756 | 2005 | cardiovascular disease | cardiovascular disease (9.36, 0.005); egg quality (9.36, 0.005); prebiotics (5.81, 0.05); absorption (5.74, 0.05); human (5.74, 0.05) |
| Representative keywords: metabolism, fatty acid, supplementation, fructo oligosaccharide, oligosaccharide, absorption, glucose, nmr spectroscopy, cell, cardiovascular disease | | | | | |
| 6 | 26 | 0.786 | 2007 | diversity | diversity (22.77, 1.0E-4); expression (22.77, 1.0E-4); inflammation (9.27, 0.005); gene (9.07, 0.005); cloning (9.07, 0.005) |
| Representative keywords: identification, expression, health, diversity, acid, obesity, gradient gel electrophoresis, crohns disease, inflammation, induced obesity | | | | | |
| 7 | 20 | 0.884 | 2003 | cellular fatty acids | cellular fatty acids (9.91, 0.005); infant (8.25, 0.005); piglet (6.81, 0.01); host-microbe cross-talk (6.81, 0.01); genetic factors (6.81, 0.01) |
| Representative keywords: growth, diet, organic acid, cellular fatty acid, children, apparent digestibility,, lactobacilli, rheumatoid arthriti, culture, gas liquid, chromatography | | | | | |
| 8 | 18 | 0.852 | 2005 | equol | equol (22.68, 1.0E-4); daidzein (18.43, 1.0E-4); intestinal microorganisms (6.11, 0.05); historical (6.11, 0.05); phytoestrogens (6.11, 0.05) |
| Representative keywords: gut microflora, lipid peroxidation, bioavailability, genistein, binding, phytoestrogen, estrogen, systems biology, increases fecal, bifidobacteria, phyto estrogen | | | | | |

**Supplementary Table 3** Cluster information of keywords network (2011-2020)

| Cluster ID | Size | Silhouette | Mean (year) | Cluster label | Top Terms (log-likelihood ratio, p-level) |
| --- | --- | --- | --- | --- | --- |
| 0 | 162 | 0.7 | 2014 | insulin resistance | insulin resistance (132.81, 1.0E-4); obesity (105.69, 1.0E-4); non-alcoholic fatty liver disease (75.99, 1.0E-4); nonalcoholic fatty liver disease (75.39, 1.0E-4); lipid metabolism (68.76, 1.0E-4) |
| Representative keywords: gut microbiota, insulin resistance, obesity, inflammation, lipid metabolism, oxidative stress, expression, metabolic syndrome, mice, diet induced obesity, bile acid, adipose tissue, acid, high fat diet, mechanism | | | | | |
| 1 | 138 | 0.694 | 2013 | diversity | diversity (78.81, 1.0E-4); short-chain fatty acids (68.64, 1.0E-4); short-chain fatty acid (65.45, 1.0E-4); insulin resistance (57.55, 1.0E-4); dietary fiber (55.29, 1.0E-4) |
| Representative keywords: intestinal microbiota, chain fatty acid, metabolism, health, diet, bacteria, short-chain fatty acid, cholesterol, fatty acid, dietary fiber, diversity, probiotics, impact, gut microbiome, microbiota | | | | | |
| 2 | 117 | 0.534 | 2017 | inflammatory bowel disease | inflammatory bowel disease (157.72, 1.0E-4); crohns disease (89.68, 1.0E-4); intestinal inflammation (58.11, 1.0E-4); ulcerative colitis (54.53, 1.0E-4); alzheimers disease (52.53, 1.0E-4) |
| Representative keywords: inflammatory bowel disease, ulcerative coliti, polyunsaturated fatty acid, lipid peroxidation, crohns disease, homeostasis, mouse model, brain, innate immunity, immune system, regulatory t cell, inflammatory response, t cell, sodium butyrate, irritable bowel syndrome | | | | | |
| 3 | 115 | 0.713 | 2015 | growth performance | growth performance (206.23, 1.0E-4); broiler (125.91, 1.0E-4); performance (105.69, 1.0E-4); broilers (97.72, 1.0E-4); poultry (83.73, 1.0E-4) |
| Representative keywords: growth performance, supplementation, in vitro, intestinal microflora, immune response, gastrointestinal tract, performance, lactic acid bacteria, extract, polyphenol, gut microflora, nutrient digestibility, lactobacillus, antioxidant, antioxidant activity | | | | | |
| 4 | 114 | 0.692 | 2016 | cardiovascular disease | cardiovascular disease (145.61, 1.0E-4); blood pressure (60.59, 1.0E-4); double blind (52.79, 1.0E-4); atherosclerosis (47.04, 1.0E-4); trimethylamine-n-oxide (44.27, 1.0E-4) |
| Representative keywords: cardiovascular disease, risk, fecal microbiota, double blind, blood pressure, consumption, lipid profile, atherosclerosis, trimethylamine n oxide, serum, coronary heart disease, type 2 diabetes mellitus, nutrition, body mass index, lactobacillus acidophilus | | | | | |
| 5 | 82 | 0.57 | 2017 | mass spectrometry | mass spectrometry (41.88, 1.0E-4); metabolomics (31.6, 1.0E-4); disease (31.04, 1.0E-4); plasma (27.58, 1.0E-4); profile (26.16, 1.0E-4) |
| Representative keywords: disease, plasma, profile, cancer, mass spectrometry, biomarker, sp nov, high fat, leptin, metabolomics, dysfunction, cholesterol metabolism, early life, tissue, progression | | | | | |
| 6 | 12 | 0.963 | 2015 | flavobacteriaceae | flavobacteriaceae (37.1, 1.0E-4); family (37.1, 1.0E-4); reclassification (37.1, 1.0E-4); mussel (37.1, 1.0E-4); emended description (37.1, 1.0E-4) |
| Representative keywords: gen. Nov, 16s rrna gene, serum metabolome, emended description, genetics, ph, chromatography, family, reclassification, mussel, mytilus coruscus, flavobacteriaceae | | | | | |
| 7 | 8 | 0.962 | 2018 | larval zebrafish | larval zebrafish (18.26, 1.0E-4); chlorpyrifos (18.26, 1.0E-4); toxicity (17.93, 1.0E-4); metabolic disorder (9.39, 0.005); murine model (9.12, 0.005) |
| Representative keywords: exposure, toxicity, disorder, behavior, water, dietary, nanoparticle, ingestion | | | | | |

**Supplementary Table 4** Cluster information of keywords network (2021-2024)

| Cluster ID | Size | Silhouette | Mean (year) | Cluster label | Top Terms (log-likelihood ratio, p-level) |
| --- | --- | --- | --- | --- | --- |
| 0 | 124 | 0.74 | 2021 | growth performance | growth performance (277.73, 1.0E-4); intestinal microbiota (173.1, 1.0E-4); obesity (143.29, 1.0E-4); growth (128.69, 1.0E-4); broiler (112.39, 1.0E-4) |
| 1 | 121 | 0.525 | 2021 | gut-brain axis | gut-brain axis (82.63, 1.0E-4); gut microbiome (82.5, 1.0E-4); parkinsons disease (60.44, 1.0E-4); alzheimers disease (58.66, 1.0E-4); depression (55.94, 1.0E-4) |
| 2 | 113 | 0.608 | 2021 | functional food | functional food (37.84, 1.0E-4); homeostasis (32, 1.0E-4); extract (31.35, 1.0E-4); antioxidant activity (29.67, 1.0E-4); functional foods (29.2, 1.0E-4) |
| 3 | 92 | 0.663 | 2021 | atherosclerosis | atherosclerosis (127.62, 1.0E-4); cardiovascular disease (109.98, 1.0E-4); trimethylamine n-oxide (80.26, 1.0E-4); coronary artery disease (61.16, 1.0E-4); hypertension (60.16, 1.0E-4) |
| 4 | 89 | 0.64 | 2021 | probiotics | probiotics (68.47, 1.0E-4); type 2 diabetes mellitus (64.07, 1.0E-4); lipid profile (56.75, 1.0E-4); type 2 diabetes (55.04, 1.0E-4); gestational diabetes mellitus (42.19, 1.0E-4) |
| 5 | 85 | 0.873 | 2021 | gut microbiota | gut microbiota (146.37, 1.0E-4); obesity (70.1, 1.0E-4); lipid metabolism (68.68, 1.0E-4); high-fat diet (55.5, 1.0E-4); short-chain fatty acids (41.36, 1.0E-4) |
| 6 | 83 | 0.691 | 2021 | bile acids | bile acids (101.17, 1.0E-4); bile acid (98.13, 1.0E-4); nonalcoholic fatty liver disease (94.99, 1.0E-4); farnesoid x receptor (90.83, 1.0E-4); non-alcoholic fatty liver disease (86.05, 1.0E-4) |
| 7 | 67 | 0.739 | 2021 | ulcerative colitis | ulcerative colitis (129.9, 1.0E-4); inflammatory bowel disease (111.93, 1.0E-4); crohns disease (55.92, 1.0E-4); fecal microbiota (42.1, 1.0E-4); dendritic cell (37.38, 1.0E-4) |
| 8 | 44 | 0.791 | 2021 | 16s rrna | 16s rrna (60.93, 1.0E-4); 16s rrna gene sequencing (39.97, 1.0E-4); serum metabolome (38.6, 1.0E-4); gut bacteria (35.63, 1.0E-4); diversity (29.96, 1.0E-4) |
| 9 | 11 | 0.955 | 2021 | cadmium | cadmium (27.82, 1.0E-4); hepatotoxicity (26.51, 1.0E-4); microplastics (24.92, 1.0E-4); stereoisomeric selectivity (19.88, 1.0E-4); cypermethrin (19.88, 1.0E-4) |

**Supplementary**
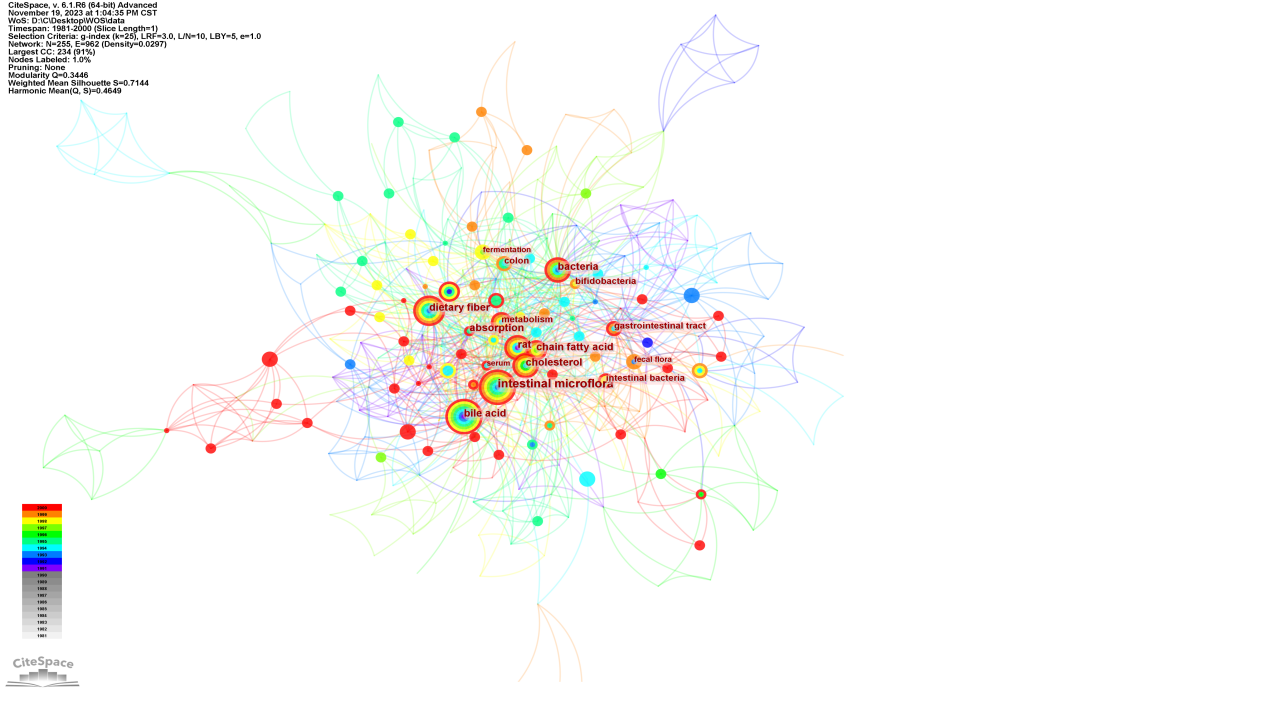
 **Figure 1** the network of keywords (1981-2000)

**Supplementary**
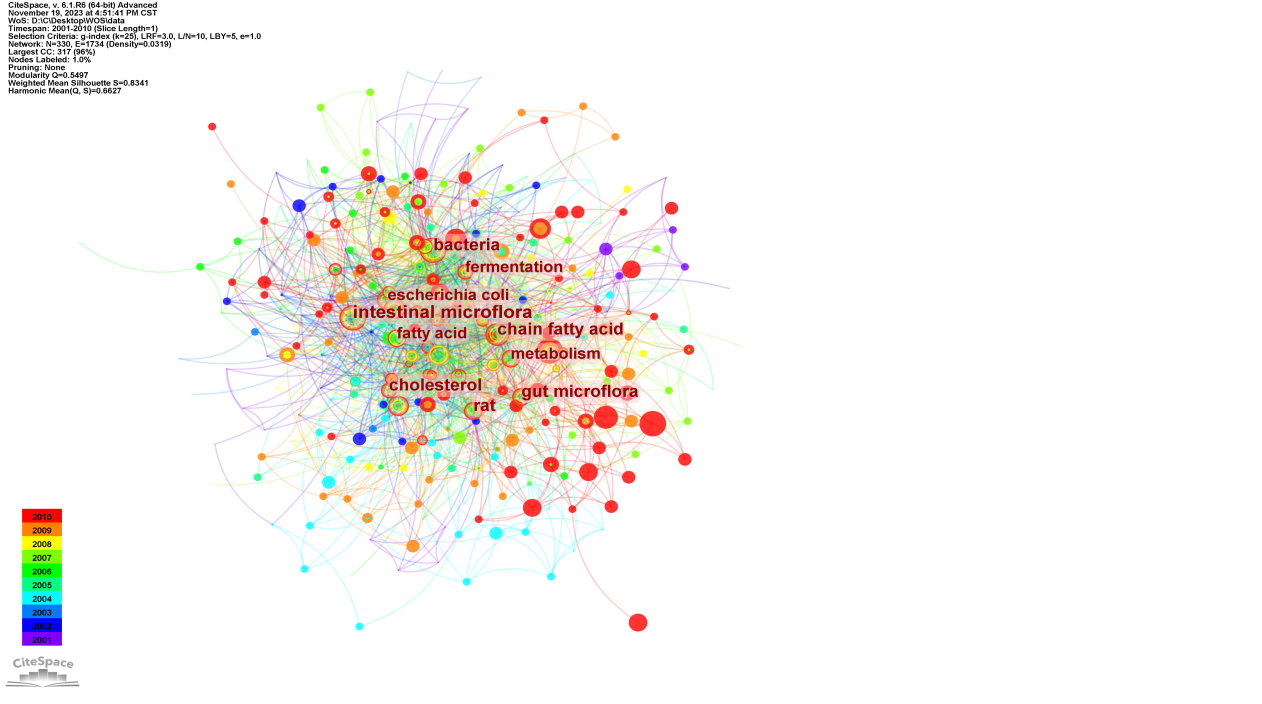
 **Figure 2** the network of keywords (2001-2010)

**Supplementary
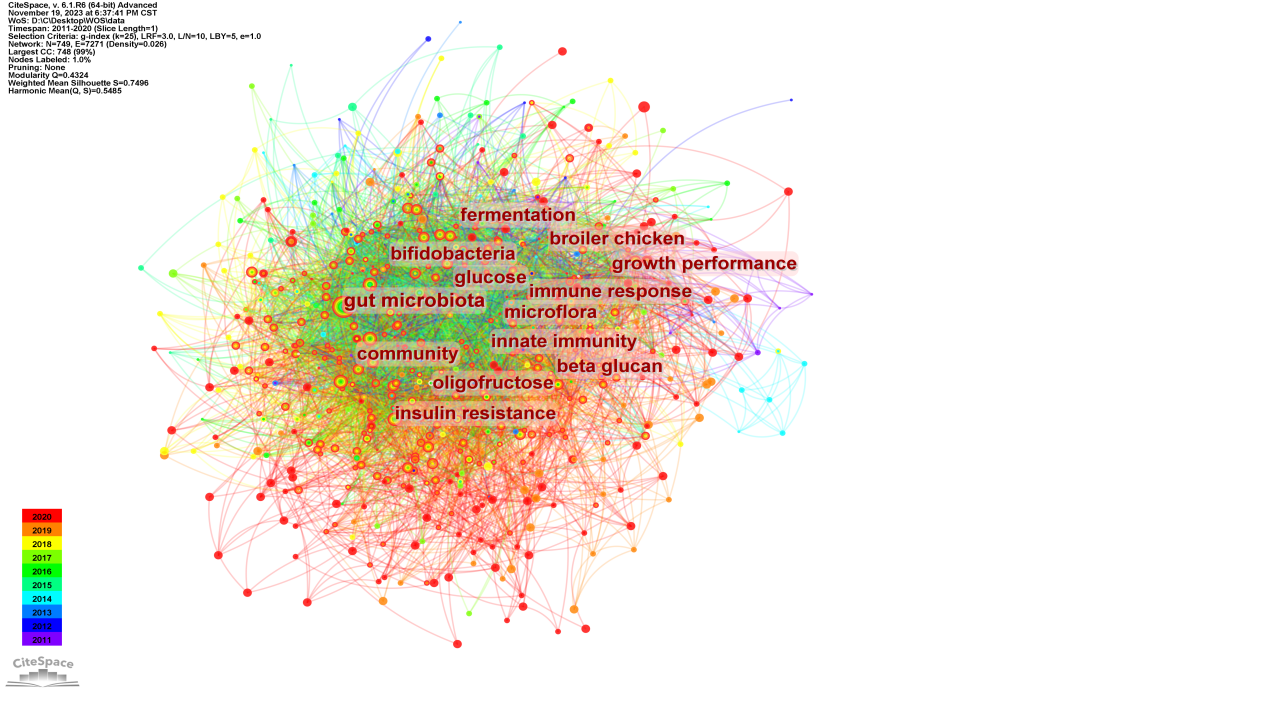
 Figure 3** the network of keywords (2011-2020)

**Supplementary Figure 4** the network of keywords (2021-2024)
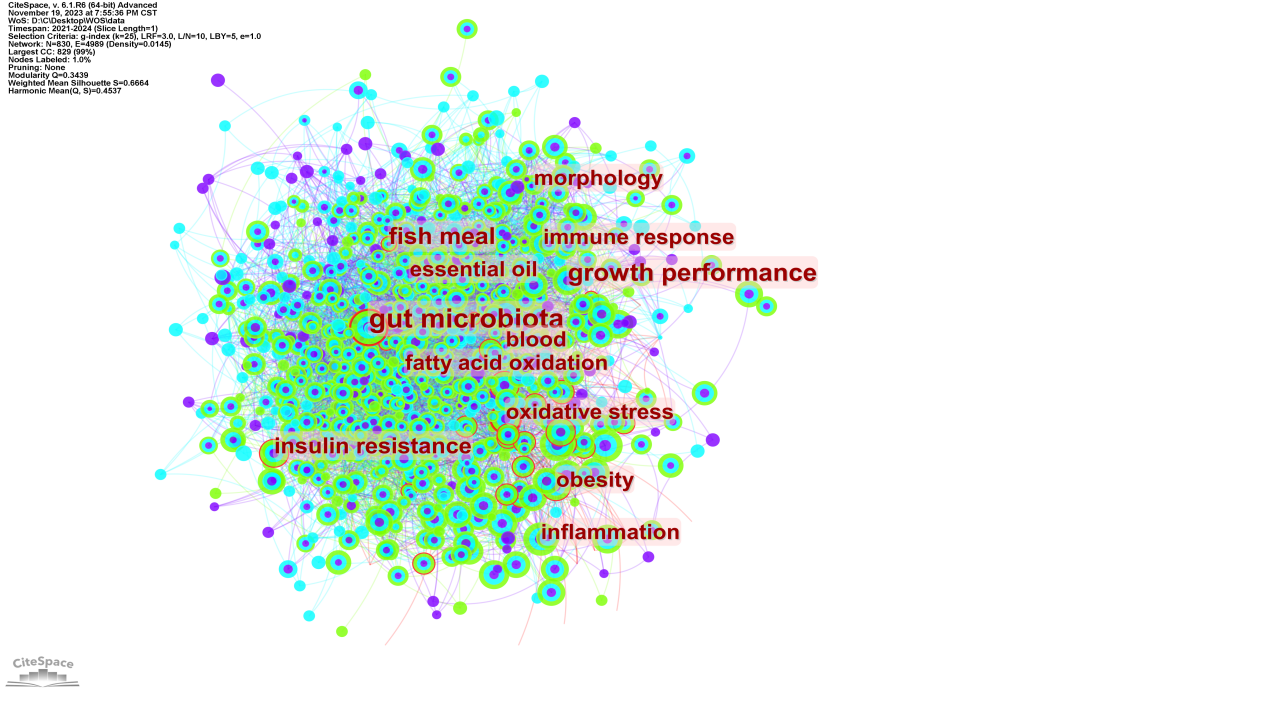

Supplement: Supplementary file 1 [file Table_1.DOCX]
